# Enhanced dominance of soil moisture stress on vegetation growth in Eurasian drylands

**DOI:** 10.1093/nsr/nwad108

**Published:** 2023-04-24

**Authors:** Yu Zhang, Yangjian Zhang, Xu Lian, Zhoutao Zheng, Guang Zhao, Tao Zhang, Minjie Xu, Ke Huang, Ning Chen, Ji Li, Shilong Piao

**Affiliations:** Key Laboratory of Ecosystem Network Observation and Modeling, Institute of Geographic Sciences and Natural Resources Research, Chinese Academy of Sciences, Beijing 100101, China; College of Resources and Environment, University of Chinese Academy of Sciences, Beijing 100190, China; Key Laboratory of Ecosystem Network Observation and Modeling, Institute of Geographic Sciences and Natural Resources Research, Chinese Academy of Sciences, Beijing 100101, China; College of Resources and Environment, University of Chinese Academy of Sciences, Beijing 100190, China; Sino-French Institute for Earth System Science, College of Urban and Environmental Sciences, Peking University, Beijing 100871, China; Department of Earth and Environmental Engineering, Columbia University, New York, NY 10027, USA; Key Laboratory of Ecosystem Network Observation and Modeling, Institute of Geographic Sciences and Natural Resources Research, Chinese Academy of Sciences, Beijing 100101, China; Key Laboratory of Ecosystem Network Observation and Modeling, Institute of Geographic Sciences and Natural Resources Research, Chinese Academy of Sciences, Beijing 100101, China; College of Agronomy, Shenyang Agricultural University, Shenyang 110866, China; College of Agronomy, Shenyang Agricultural University, Shenyang 110866, China; Key Laboratory of Ecosystem Network Observation and Modeling, Institute of Geographic Sciences and Natural Resources Research, Chinese Academy of Sciences, Beijing 100101, China; Department of Geosciences and Natural Resource Management, University of Copenhagen, Copenhagen 1350, Denmark; Key Laboratory of Wetland Ecology and Environment, Northeast Institute of Geography and Agroecology, Chinese Academy of Sciences, Changchun 130102, China; Key Laboratory of Ecosystem Network Observation and Modeling, Institute of Geographic Sciences and Natural Resources Research, Chinese Academy of Sciences, Beijing 100101, China; Department of Geography, School of Geography and Information Engineering, China University of Geosciences, Wuhan 430078, China; Sino-French Institute for Earth System Science, College of Urban and Environmental Sciences, Peking University, Beijing 100871, China; State Key Laboratory of Tibetan Plateau Earth System, Resources and Environment, Institute of Tibetan Plateau Research, Chinese Academy of Sciences, Beijing 100085, China

**Keywords:** soil water content, vapor pressure deficit, vegetation growth, decoupling, non-linear relation

## Abstract

Despite the mounting attention being paid to vegetation growth and their driving forces for water-limited ecosystems, the relative contributions of atmospheric and soil moisture dryness stress on vegetation growth are an ongoing debate. Here we comprehensively compare the impacts of high vapor pressure deficit (VPD) and low soil water content (SWC) on vegetation growth in Eurasian drylands during 1982–2014. The analysis indicates a gradual decoupling between atmospheric dryness and soil dryness over this period, as the former has expanded faster than the latter. Moreover, the VPD–SWC relation and VPD–greenness relation are both non-linear, while the SWC–greenness relation is near-linear. The loosened coupling between VPD and SWC, the non-linear correlations among VPD–SWC-greenness and the expanded area extent in which SWC acts as the dominant stress factor all provide compelling evidence that SWC is a more influential stressor than VPD on vegetation growth in Eurasian drylands. In addition, a set of 11 Earth system models projected a continuously growing constraint of SWC stress on vegetation growth towards 2100. Our results are vital to dryland ecosystems management and drought mitigation in Eurasia.

## Teaser

This study reveals a gradually decoupled relationship between atmospheric and soil moisture dryness in Eurasian drylands. As time goes by, the relative influential magnitude of soil moisture dryness stress on dryland ecosystems strengthens, while that of atmospheric dryness weakens and this relative dominance will persist into the future warming.

## INTRODUCTION

Drylands (i.e. the extent of arid climate) account for ∼42% of the global land surface and support more than one-third of the world's population [1]. Drylands are characterized by permanent or seasonal water deficiency [1]. Besides being the habitat for ∼30% of the world's endangered and endemic species [2], drylands provide a range of social benefits for human beings [3]. As one of the most vulnerable ecosystems on Earth [4], drylands face the risk of expansion and ecological degradation due to water deficiency, sometimes manifested as a systemic or abrupt state change [5]. As the two most common types of dryness, the relative dominance of high vapor pressure deficit (VPD) and low soil water content (SWC) on vegetation growth is highly relevant to dryland ecosystems restoration.

**Figure 1.**
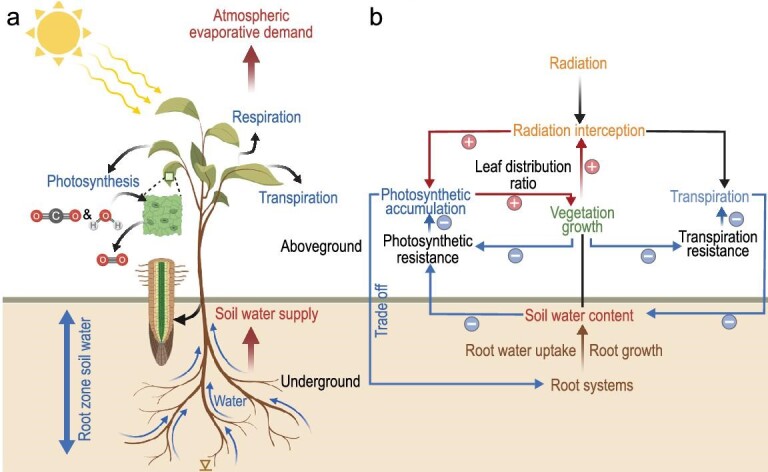
Conceptual illustration of plant-centric interpretation of vegetation water stress through the pathways of atmospheric evaporative demand and soil water supply.

Droughts (i.e. meteorological water-shortage conditions) are the most widespread and influential stressors on vegetation in drylands [6,7]. According to the soil–plant–atmosphere-continuum (SPAC) theory, vegetation growth is physiologically regulated by a balance between water supply and water demand. Water supply refers to the available SWC (from field capacity to wilting point) that plants can absorb by the roots, while water demand is driven by VPD that forces plant water into the atmosphere through leaf stomata [8] (Fig. 1a). During drought events, vegetation senses water stress from both high atmospheric water demand and limited water supply in the soil reservoir [9]. Both factors have impacts on vegetation growth in the literature (Supplementary Table S1), but there is still an ongoing debate in terms of the relative dominance of VPD versus SWC stress, especially in drylands under a changing climate [10]. Moreover, the temporal shift of the dominant water stress effect between SWC and VPD, as we investigate here, has rarely been studied (Supplementary Table S1).

The effects of SWC and VPD on vegetation growth are associated with two mechanisms, i.e. carbon starvation and hydraulic failure. On the one hand, VPD has been extensively reported to regulate global vegetation growth [11]. High VPD typically drives plants to partially close their stomata to reduce water loss and avoid critical water tension within the xylem [12] (Fig. 1a), which presents strong physiological limitations on leaf photosynthesis and vegetation growth. Under extremely high VPD, leaf stomata are completely closed and transpiration would be close to zero [13]. However, previous findings regarding VPD effects on vegetation may be overestimated [9] due to their overlooking plant stomatal acclimation to a decade-long persistent increasing VPD [8]. Mounting evidence points to more comprehensive effects of elevated VPD on plant physiology, also on the anatomical [14,15], biochemical [16] and evolution [17] processes, but these conclusions are mostly isolated from variations in SWC [18]. On the other hand, increased leaf area reduces canopy resistance [19] and causes an increase in plant transpiration. Being the major water source for plant use, SWC is likely to decrease as transpiration increases, which constrains subsequent root water uptake [20]. Moreover, soil moisture deficit may trigger catheter embolism, stagnates water transportation [21], lowers stomatal conductance and increases photosynthetic resistance [22] (Fig. 1b).

Knowledge on the individual effects of high VPD and low SWC on vegetation growth is critical as this is related to whether the rising ecosystem water stress is mainly supply-driven or demand-driven [10]. However, it is challenging to distinguish the effects of high VPD and low SWC on vegetation growth in drylands due to the following reasons: (i) atmospheric and soil processes, such as VPD and SWC, are highly coupled at monthly and yearly timescales [23] (Supplementary Fig. S27); (ii) potential non-linear relationships exist among VPD, SWC and vegetation growth [24]; and (iii) correlation does not necessarily imply causality [25]. Due to our limited understanding, state-of-the-art terrestrial ecosystem models represent water stress on ecosystem gross primary production (GPP) either as a function of SWC only [26,27], VPD only [28–31] or occasionally their combination [32].

Previous studies have mostly focused on a global or a regional scale, in which multiple limiting factors (e.g. SWC, VPD, temperature, radiation) act interactively on vegetation growth [33] (Supplementary Table S1). In global drylands where ecosystem function is primarily constrained by water, focusing on the relatively simpler growth–water relations might boost assessment accuracies in disentangling the effects of VPD and SWC (Supplementary Table S1). Among the global drylands, Eurasia has the largest continuous extent of drylands and also supports a large human population [34]. Eurasian drylands are facing the double threats of climate change and human disturbances. Since the 1980s, the warming rate in Eurasian drylands has far exceeded the global average [35]. Extreme climates events have also increased in frequency and severity herein [36]. Terrestrial water storage has declined consistently and water scarcity risk has risen [37], leading to various degrees of ecosystem degradation [10].

Eurasian dryland ecosystems consist mainly of grassland and cropland [10], which are sensitive and fragile to environmental changes and disturbances. Most of the countries in Eurasian drylands are developing ones, with a high proportion of people living below the United Nations poverty line. Agricultural production, mainly comprising crops and livestock [1], is highly vulnerable to climate change. To protect therein dryland ecosystems, improve human livelihood and achieve the Sustainable Development Goals (SDGs) [38], the prerequisite is to clarify the longstanding question regarding which type of water stress dominates in dryland ecosystems.

In this study, we assessed long-term vegetation response to water stress during 1982–2100 in Eurasian drylands by integrating remote sensing data sets, climatic reanalysis data sets, flux and environmental measurements, terrestrial ecosystem models and Earth system model outputs (ESMs) of VPD, SWC and vegetation growth indices (see Supplementary methods and Supplementary Tables S2–S4). Among them, we applied three sets of remote sensing data sets to characterize various aspects of vegetation growth (see Supplementary methods: ‘Vegetation growth indices’), comprehensively reflecting vegetation structure, function and water status. Our 3-fold objectives were to (i) compare VPD and SWC dynamics; (ii) examine the pairwise relationship between VPD, SWC and vegetation growth; and (iii) clarify the relative importance of SWC stress versus VPD stress for vegetation growth. The related research findings are critical for advancing our understanding and improving prediction on global dryland ecosystem state changes.

## RESULTS

### The decoupled atmospheric and soil dryness

**Figure 2.**
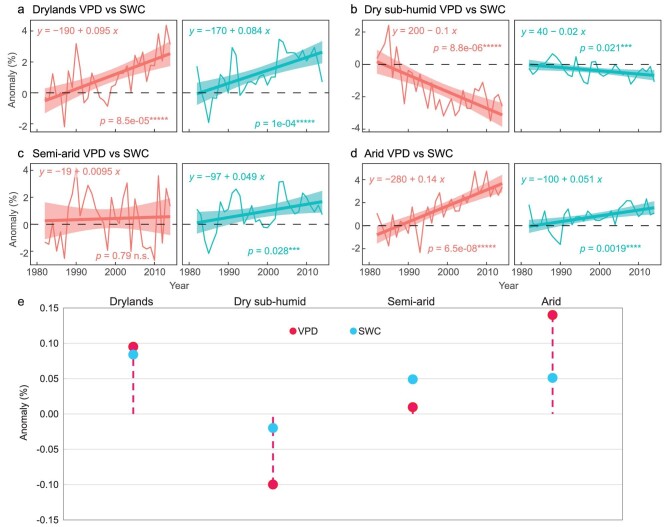
Decoupling of vapor pressure deficit (VPD, red line) and soil water content (SWC, cyan line) over Eurasian drylands. (a)–(d) Anomaly (as %) of areal fraction of drylands evaluated by vapor pressure deficit (f_atm_) and soil water content (f_soil_) during 1982–2014 for (a) Eurasian drylands, (b) dry sub-humid, (c) semi-arid and (d) arid regions. (e) Trends magnitudes of f_atm_ and f_soil_ along aridity gradient. Similar to regions with aridity index (AI) of <0.65, f_atm_ and f_soil_ are computed using threshold values of the corresponding metric. Anomalies were computed by subtracting the climatological mean of 1961–90 (a subset of years during this period, 1982–90) [1]. The shaded areas represent the 95% confidence intervals. Statistical significances are shown as symbols ‘*****’, ‘****’, ‘***’ and ‘n.s.’, denoting *p* < 0.001, *p* < 0.005, *p* < 0.01 and *p* > 0.1, respectively. ERA-Interim VPD and GLEAM SWC products were used.

Two approaches were used to compare atmospheric and soil dryness changes over Eurasian drylands (Fig. 2 and Supplementary Figs S1–S6). The first one calculates zonal average values of VPD and SWC over Eurasian drylands using a fixed drylands extent (as in Supplementary Fig. S1). The second one maps a temporally evolving drylands extent by applying a fixed threshold to each aridity metric (see Supplementary methods: ‘Drylands extent with different aridity metrics’; Supplementary Fig. S4). In the second approach, the fraction of Eurasian drylands area is denoted as f_atm_ (atmospheric aridity) and f_soil_ (soil aridity) in accordance with metrics defined by VPD and SWC, respectively.

Based on the first approach, on average, VPD in Eurasian drylands showed a significant increase from 1958 to 2014, and also for the more recent period from 1982 to 2014 (Supplementary Fig. S3a). Observed total-column (or root-zone) SWC (such as GLDAS and GLEAM) revealed a significant drying trend during 1958–2014 as well as during 1982–2014 (Supplementary Fig. S3b). The concurrent trends of increasing VPD and decreasing SWC suggested that Eurasian drylands were exposed to aggravated atmospheric and soil dryness. According to the second approach, both f_atm_ and f_soil_ have expanded significantly since 1982, with the former expanding faster (0.95% decade^–1^) than the latter (0.84% decade^–1^) (Fig. 2a). We repeated the analysis (Fig. 2a) using average by all data sets and they all corroborated the inconsistent rates of changes between VPD and SWC over Eurasian drylands (Supplementary Fig. S5). Consistently, the increasing rate of f_atm_ was faster than that of f_soil_. Neither of them was significant during 1958–2014 (Supplementary Fig. S5a), but both of them have increased significantly since the 1980s (Supplementary Fig. S5b). The difference of increasing rate (0.51% decade^–1^) between the f_atm_ and f_soil_ since 1982 was more apparent than that (0.11% decade^–1^) since 1958. It suggested the coupling between VPD and SWC became loosened over the last three decades.

We further found that the increasing rates of f_atm_ and f_soil_ varied along the gradient of background aridity levels (Fig. 2b–e). The highest increasing rate of f_atm_ (1.4% decade^–1^) was found in arid regions (Fig. 2d and e). However, f_atm_ increased slightly in semi-arid regions, where f_soil_ increased significantly (Fig. 2c and e). Both f_atm_ and f_soil_ decreased in dry sub-humid regions, with the former decreasing faster (1% decade^–1^) than the latter (0.2% decade^–1^) (Fig. 2b and e). The pattern along the aridity gradient illustrated that f_atm_ and f_soil_ exhibited distinct paces of change in transitional zones between dry and wet climates, i.e. the dry sub-humid and semi-arid regions. The average of all data sets confirmed the above findings (Supplementary Fig. S6).

### Non-linear interactions among VPD, SWC and vegetation growth

**Figure 3.**
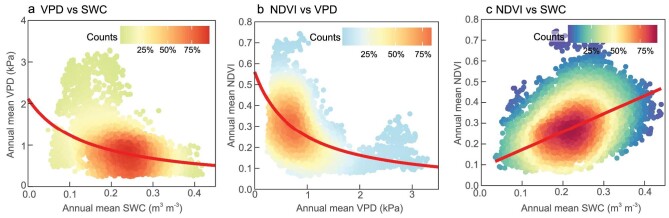
Relationships among vapor pressure deficit (VPD), soil water content (SWC) and vegetation growth over Eurasian drylands. Relationships between (a) VPD and SWC, (b) normalized difference vegetation index (NDVI) and VPD and (c) NDVI and SWC during 1982–2014. All vegetated grids in Eurasian drylands are included. ERA-Interim VPD, GLEAM SWC and GIMMIS NDVI products were used.

The decoupled atmospheric and soil dryness trends provide an opportunity to disentangle the contribution of VPD and SWC to vegetation growth. Using VPD, SWC and the normalized difference vegetation index (NDVI) of all vegetated grids in aridity index (AI)-defined drylands (Supplementary Fig. S1), we further examined their pairwise relationships over the last three decades (Fig. 3 and Supplementary Fig. S7).

The long-term relationship across space between VPD and SWC was non-linear (*p* < 0.001) (Fig. 3a). The VPD trajectory transited from being flat to steep when SWC was below ∼0.2 m^3^ m^–3^. This pattern suggested that atmosphere responded to soil dryness in two phases, exhibited as early slow response and later fast one. Equivalently, the SWC trajectory transited from being steep to flat when VPD increased to ∼0.9 kPa, which also demonstrated two phases response pattern with early quick response and a later slowed one. A non-linear relationship across space was further detected between VPD and NDVI (*p* < 0.001) (Fig. 3b). NDVI decreased at a slower rate under higher atmospheric dryness, then it leveled off when VPD increased to ∼0.9 kPa. However, NDVI decreased near-linearly with lowered SWC levels. The linear models (R = 0.51, *p* < 0.001) outperformed non-linear models in describing the relationships between NDVI and SWC (Fig. 3c). Furthermore, the non-linear relations between VPD and SWC (*p* < 0.001) (Supplementary Fig. S7a–g), VPD and NDVI (*p* < 0.001) (Supplementary Fig. S7h–j), and the near-linear relationship between NDVI and SWC (R = 0.68, *p* < 0.001) (Supplementary Fig. S7k) were all verified by the other 11 data sets.

### SWC stress dominates vegetation growth

With potential non-linearity in the complex system, the correlation between vegetation growth and water availability does not necessarily reflect a causal relationship. The percentile binning method was used to analyse the causal relationship between VPD or SWC stress and vegetation growth (see Supplementary methods: ‘Percentile binning’). In the analysis, three types of relationships were defined (see ‘Methods: Water-stressed ecosystems redefinitions’), i.e. non-water-stressed ecosystems, water-stressed ecosystems and ecosystems dominated by SWC (or VPD) stress (dominant water stress effect).

**Figure 4.**
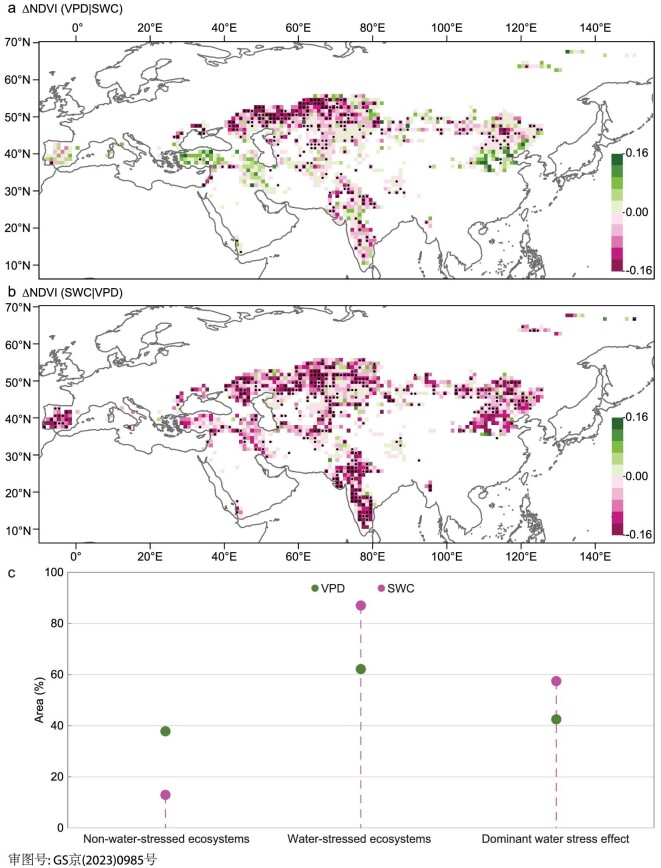
Relative roles of vapor pressure deficit (VPD) and soil water content (SWC) on normalized difference vegetation index (NDVI) over Eurasian drylands during 1982–2014. (a) and (b) Spatial distribution of the changes in NDVI caused by (a) high VPD and (b) low SWC and their dominant water stress effect (black circles; see ‘Methods: Water-stressed ecosystems redefinitions’). (c) Relative contributions of high VPD and low SWC to the variation in NDVI. ERA-Interim VPD, GLEAM SWC and GIMMIS NDVI products were used.

For non-VPD-stressed ecosystems and non-SWC-stressed ecosystems (see ‘Methods: Water-stressed ecosystems redefinitions’), the areal fraction of the former (38%, Fig. 4a and c) was larger than that of the latter (13%, Fig. 4b and c). The strongly positive values for ΔNDVI (VPD|SWC) were found in Spain, Turkey, Iran and the North China Plain (Fig. 4a), whereas weakly positive values for ΔNDVI (SWC|VPD) were scattered in Central Asia and Northwest China (Fig. 4b). Correspondingly, the area of SWC-stressed ecosystems (87%, Fig. 4b and c) was larger than that of VPD-stressed ecosystems (62%, Fig. 4a and c), indicating that SWC placed strong constraints on vegetation growth in Eurasian drylands. The negative value for ΔNDVI (VPD|SWC) was close to 0 in Central Asia, Northeast China and Tibet Plateau (Fig. 4a) while that for ΔNDVI (SWC|VPD) was low across most vegetated land areas (Fig. 4b). Meanwhile, the dominant role of SWC stress on vegetation growth was further verified by six out of seven of the included data sets (see Supplementary methods: ‘Drought indices’; Supplementary Figs S8–S14). Further, we identified the dominant water stress effect as the stressor that had a greater suppressive effect on vegetation growth when compound water stress effects exist (see ‘Methods: Water-stressed ecosystems redefinitions’). The ecosystems dominated by SWC stress was slightly larger than that dominated by VPD stress (black circles in Fig. 4). The former was distributed primarily in South Europe, Central Asia, East Asia and India (black circles in Fig. 4b). Also, the larger dominant stress area for SWC was robust to six out of seven of the included data sets (see Supplementary methods: ‘Drought indices’; Supplementary Figs S8–S14).

Besides the percentile binning approach, we also performed partial correlation to distinguish individual effects of VPD and SWC (see ‘Percentile binning’ from Supplementary methods: ‘Spearman partial correlation’; Supplementary Figs S15 and S16). Given the opposite sign between SWC and VPD, the partial correlation between NDVI and SWC is displayed in the number opposite the actual value. On the one hand, most negative correlation areas between NDVI and VPD (or SWC) (Supplementary Fig. S15) coincided with water-stressed ecosystems (Fig. 4a and b). A negative correlation indicates that vegetation was less productive under drier conditions (Supplementary Fig. S3) than under normal conditions, suggesting that permanent or seasonal water deficit can suppress vegetation growth. On the other hand, a significant negative correlation between NDVI and VPD (or SWC) (*p* < 0.05) implies a strong water stress effect placed by VPD (or SWC). The distribution of significant negative correlation (white circles in Supplementary Fig. S15) was almost consistent with areas dominated by VPD (or SWC) stress (black circles in Fig. 4a and b). Moreover, the relationship between NDVI and SWC showed stronger negative correlations for all of the included data sets (Supplementary Fig. S16). We also repeated the analyses using other two metrics of vegetation growth, namely the microwave-based vegetation optical depth (VOD) and the NIRv-based GPP. The analysis linking VOD to VPD and SWC further confirmed the above results for both percentile binning (Supplementary Fig. S17) and partial correlation (Supplementary Fig. S19a and b) methods. The GPP-based analysis also confirmed the above results for the partial correlation method (Supplementary Fig. S19c and d). However, for the binning approach, ecosystems with VPD stress on GPP (71%, Supplementary Fig. S18a and c) had a slightly larger area than those with SWC stress (67%, Supplementary Fig. S18b and c), indicating an equally important role for SWC and VPD on ecosystem GPP during 1982–2014. Similarly, the areal fraction with VPD as the dominant stressor on ecosystem GPP (59%) was larger than that with SWC as the dominant stressor (41%) (black circles in Supplementary Fig. S18a and b).

**Figure 5.**
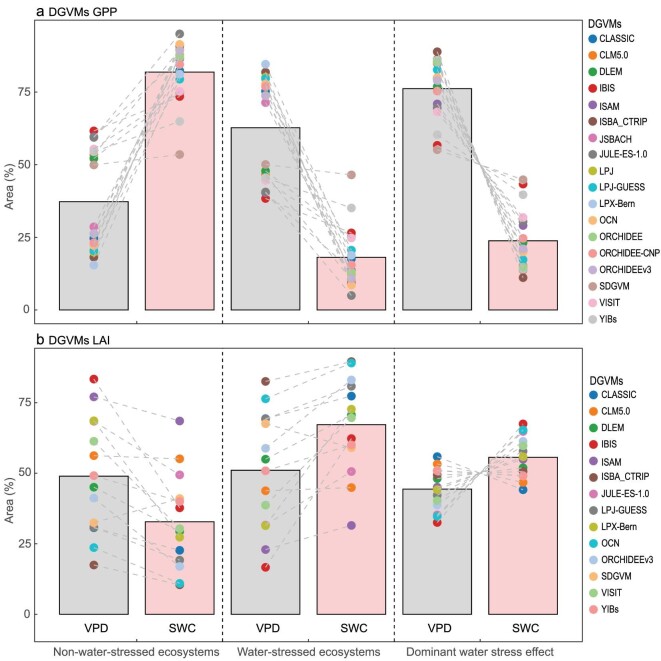
Relative roles of vapor pressure deficit (VPD) and soil water content (SWC) on gross primary production (GPP) or leaf area index (LAI) over Eurasian drylands during 1982–2014. (a) 18 GPP and (b) 14 LAI simulations from dynamic global vegetation models (DGVMs) of the ‘Trends in net land-atmosphere carbon exchange’ (TRENDY). For TRENDY DGVMs, S3 simulations were used, including changing climate forcing, rising atmospheric CO_2_ concentrations and land-use change. The bars represent their median. ERA-Interim VPD and GLEAM SWC products were used.

In addition, we also used the same method to compare the 18 GPP simulations from dynamic global vegetation models (DGVMs) of the ‘Trends in net land-atmosphere carbon exchange’ (TRENDY) project (see Supplementary methods: ‘Percentile binning’; Supplementary Table S3) with results from satellite GPP (Fig. 5a). None of the 18 GPP models captured SWC dominance (Fig. 5a). Although there is no NDVI information in the TRENDY DGVMs, we also compared the 14 leaf area index (LAI) simulations with results from satellite NDVI (Fig. 5b). Thirteen out of the 14 models that simulated LAI captured the dominant role of SWC stress on vegetation growth (Fig. 5b). Moreover, the ecosystems dominated by SWC stress on LAI was larger than that by VPD stress for 11/14 models (Fig. 5b).

**Figure 6.**
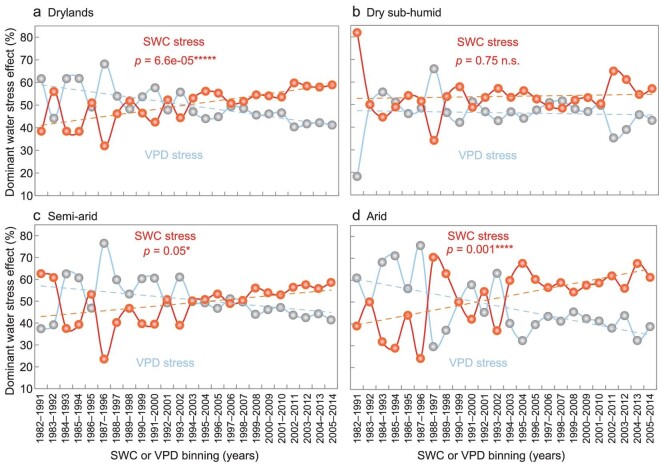
Temporal dynamics of dominant water stress effect over Eurasian drylands during 1982–2014. The trends of the dominant type of water stress effect for 24 10-year moving windows over (a) Eurasian drylands, (b) dry sub-humid, (c) semi-arid and (d) arid regions are shown. The blue line represents the ecosystems dominated by vapor pressure deficit (VPD) stress; the red line indicates the ecosystems dominated by soil water content (SWC) stress (see ‘Methods: Water-stressed ecosystems redefinitions’). SWC or VPD bins are characterized by the 10-year moving windows. Statistical significances are indicated by symbols ‘*****’, ‘****’, ‘*’ and ‘n.s.’, meaning *p* < 0.001, *p* < 0.005, *p* < 0.1 and *p* > 0.1, respectively. ERA-Interim VPD, GLEAM SWC and GIMMIS NDVI products were used.

Using a percentile binning method combined with 10-year moving windows, the temporal shift of the dominant water stress effect between SWC and VPD has been clarified (Fig. 6). The ecosystems dominated by SWC stress expanded in Eurasian drylands from 1982 to 2014 (see ‘Methods: Water-stressed ecosystems redefinitions’; Fig. 6a). Along the aridity gradient, the ecosystems dominated by SWC stress expanded fastest in arid regions, followed by semi-arid regions (Fig. 6b–d). Analysis using the average of multiple data sets confirmed the above findings, especially for arid regions (Supplementary Fig. S20). In addition, although the patterns along the aridity gradient varied for different vegetation indies, the ecosystems with SWC as the dominant stressor on VOD and GPP has also expanded slightly over Eurasian drylands since the 1980s (Supplementary Figs S21 and S22).

### Future enhanced dominance of SWC stress on vegetation growth

**Figure 7.**
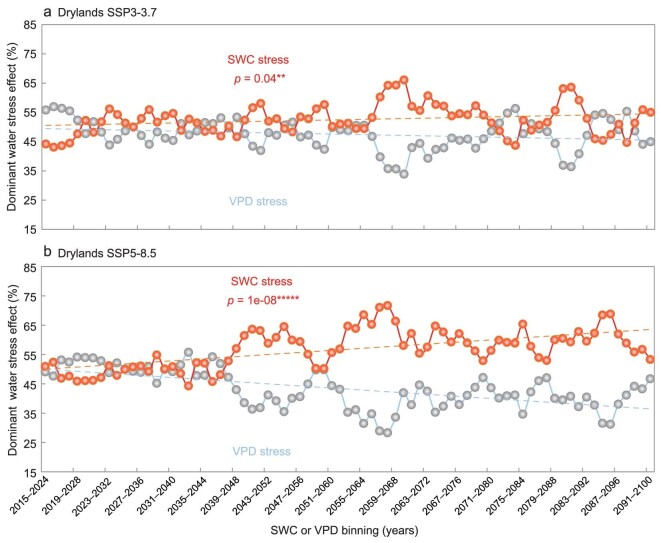
Temporal dynamics of dominant water stress effect over Eurasian drylands during 2015–2100 under (a) SSP3-7.0 and (b) SSP5-8.5 scenarios. The trends of the dominant type of water stress effect for 77 10-year moving windows over Eurasian drylands. The blue line stands for the ecosystems dominated by VPD stress; the red line indicates the ecosystems dominated by SWC stress; SWC or VPD bins are characterized by the 10-year moving windows. Statistical significances are shown as symbols ‘*****’ and ‘**’, denoting *p* < 0.001 and *p* < 0.05, respectively.

Finally, the spatio-temporal patterns of the water stressor (high VPD or low SWC) on vegetation growth were projected by the 11 ESMs under both SSP3-7.0 and SSP5-8.5 scenarios (Supplementary methods: ‘Earth system model outputs’; Supplementary Figs S23–S26 and Fig. 7).

Supplementary Fig. S23 shows spatial patterns of the multi-model mean water stress effect on GPP towards the end of this century (2015–2100) under SSP3-7.0 and SSP5-8.5 scenarios. The negative ΔGPP (SWC${|}$VPD) was low across most vegetated land areas, especially in India and the North China Plain (Supplementary Fig. S23b and d) but weakly negative values for ΔGPP (VPD${|}$SWC) were scattered in South Europe, Central Asia and India under both scenarios (Supplementary Fig. S23a and c). In general, the area of SWC-stressed ecosystems (Supplementary Fig. S23b and d) was much larger than that of VPD-stressed ecosystems (Supplementary Fig. S23a and c) across >83% of land vegetated areas under SSP3-7.0 and SSP5-8.5 scenarios, indicating that SWC stress would persistently dominate Eurasian drylands vegetation growth throughout the twenty-first century. Similarly, the ecosystems dominated by SWC stress were larger than those dominated by VPD stress (Supplementary Fig. S23). The former were mainly distributed in Central Asia and India (black circles in Supplementary Fig. S23b and d). The results were also supported by partial correlation analysis (Supplementary Fig. S24). Moreover, the ecosystems dominated by SWC stress over Eurasian drylands will expand significantly by 2100 under both SSP3-7.0 and SSP5-8.5 scenarios (Fig. 7) and the worst-case scenarios will lead to an even greater expansion for the drylands (Fig. 7b), as well as for dry sub-humid, semi-arid and arid regions (Supplementary Figs S25 and S26).

## DISCUSSION

This study revealed a progressive decoupling between atmospheric and soil water stress on dryland vegetation and the potential non-linear relationship between VPD, SWC and vegetation growth. By disentangling the causal effects of the two types of water stressors on vegetation growth, we clarified the dominance of SWC stress on vegetation growth over VPD stress across Eurasian drylands from 1982 to 2014. Specifically, the dominant effect of SWC stress on vegetation growth was projected to further increase with future warming.

It is generally believed that VPD and SWC are closely coupled on monthly to yearly timescales [23] (Supplementary Fig. S27). However, highly inconsistent trends between VPD and SWC emerge on multi-decadal or longer temporal scales for global drylands [1]. It is also predicted that historical trends of strongly increased f_atm_, and slightly increased f_soil_ will continue until the 2090s over Eurasian drylands [1]. This phenomenon is probably because each component of the SPAC (Fig. 1) has a distinct response pattern to rising atmospheric CO_2_ [1]. CO_2_ fertilization increases photosynthesis, LAI and transpiring biomass, while also reducing stomatal conductance and decreasing transpiration per unit leaf area [1,10]. Besides the physiological mechanisms behind vegetation response to CO_2_ increase [1], we will discuss the underlying physical mechanisms as follows.

First, warming alters thermodynamic processes by increasing atmospheric demand for water in drylands [39] (Supplementary Fig. S28), while leaving a relatively weaker signal of soil water deficits. Second, the decoupling of VPD and SWC is also linked to atmospheric circulation. Large-scale atmospheric circulation affects VPD and SWC by altering temperature, humidity and pressure gradients. For instance, under global warming, the Hadley circulation has strengthened and expanded, shifting the Intertropical Convergence Zone (ITCZ) northward and expanding the subtropical drylands poleward [40]. These changes can raise temperature, lower relative humidity, decrease precipitation and increase evaporation in the Eurasian drylands [41,42]. The Ferrel circulation has also strengthened and expanded under global warming, which accordingly intensifies and widens the westerlies, further increasing temperature and evaporation in the Eurasian drylands [43]. Third, local soil moisture also affects atmospheric aridity. SWC depletion due to warming lowers evaporative cooling and raises sensible heat flux, resulting in higher air temperature and lower humidity, which further increase VPD [1,44,45]. The soil moisture–atmosphere dryness feedback can amplify atmospheric aridity. The feedback can be stronger in drylands than in humid regions because of the limited water supply and high evaporative demand [46]. However, other factors such as land-use change, irrigation practices, surface albedo, cloudiness, wind speed and large-scale circulation can also modulate this feedback.

Using the space-for-time approach, we found that atmosphere over Eurasian drylands is drying faster than soil and the trajectory becomes steeper when SWC drops below a certain threshold (Fig. 3a and Supplementary Fig. S7a–g). Conversely, soil is drying slower as the atmosphere becomes drier and the trajectory becomes flat when VPD reaches a certain threshold during 1982–2014 (Fig. 3a and Supplementary Fig. S7a–g). The non-linear relation between VPD and SWC is consistent with field observations and experiments [47]. Moreover, atmospheric dryness affects vegetation growth in a non-linear manner, while soil dryness influences vegetation growth in a nearly linear way (Fig. 3b and c, and Supplementary Fig. S7h–k). Physiologically, plants reduce water loss (transpiration) from leaves by partially closing stomata under high VPD conditions, which also lowers the photosynthetic rate and leads to a leveling-off plant response above a certain high VPD value [48]. Transpiration is more resilient to VPD change [49]. Hence, NDVI decreases slower with continued atmospheric dryness and becomes more resilient when VPD exceeds a certain threshold.

A global study integrating 57 flux observations showed a non-linear relationship between SWC and NDVI [33] (Supplementary Table S1) but these flux sites are mainly located in the humid region. We found that the effect of SWC stress on NDVI follows a nearly linear pattern in Eurasian drylands. This may also be due to the specific physiological mechanism for dryland ecosystems, where vegetation transpiration has adapted to a long-term water-limited regime under soil drying conditions [46,50]. Dryland ecosystems tend to maintain a minimum threshold of SWC-limiting transpiration through sustained SWC extraction and transport by xylem [46].

Unlike previous studies that reported systemic and abrupt changes in multiple ecosystem attributes due to aridification based on AI [1], the present study utilized VPD and SWC simultaneously. AI could be problematic in depicting surface aridity changes [1] because potential evaporation is based on non-water-limited theory, while VPD and SWC should reflect the water constraint conditions more comprehensively.

The overwhelming effects of SWC stress can be explained by several mechanisms. First, VPD affects vegetation growth mainly by regulating stomata, whereas SWC regulates state changes of vegetation in drylands [51] by adjusting the valves relevant to physiological responses from the scale of individual plants to the entire ecosystems [52] (Supplementary Fig. S29). Second, Eurasian drylands have a high proportion of irrigated and rain-fed cropland (53.8%; Supplementary Fig. S30) and they face a higher risk of SWC stress than other ecosystems (Supplementary Fig. S31). Cropland in India and North China Plain mostly grows under irrigation (Supplementary Fig. S30). Under the urgent need for water saving, irrigation water may decrease [53], then the importance of SWC stress on irrigated cropland becomes more apparent, especially irrigated wheat and rice in India (Supplementary Figs S30 and S31b). Rain-fed cropland that depends on natural precipitation is largely distributed in Spain, Turkey, Iran and Central China (Supplementary Fig. S30). The increasing atmospheric CO_2_ can enhance the sensitivity of dryland vegetation to precipitation [54], then the importance of SWC stress on rain-fed cropland also becomes more apparent. Grassland is the second most prevalent ecosystem in Eurasian drylands after cropland [10] and it faces a lower risk of SWC stress than other ecosystems (Supplementary Fig. S31a). Based on 11 years (2012–22) of flux data at Naqv site (see Supplementary methods: ‘Flux and environmental measurements’), SWC dominates alpine meadow ecosystem (AME) across hourly to yearly scales (Supplementary Table S5). Furthermore, VPD-stressed AME occurs at about 9:00 and 12:00–14:00, while the AME dominated by VPD only occurs at 14:00 (Supplementary Table S6). Third, environmental lag effects play a significant role in drylands. Compared with VPD, which affects vegetation growth in relatively short periods, SWC exerts a long-lasting lagged effect [47]. In drylands, environmental lag effects are mainly caused by water infiltration and residence time [55], hormonal signaling of plants [56] and phenology [57], fine-root production and feedback on water and nutrient uptake [58], and microbial activity and potential community change [59]. All these processes can influence vegetation growth by regulating SWC and also leave plants ample time to adapt or acclimate to changes in SWC (Supplementary Fig. S32), while stomata respond to VPD in near real time [55]. Lag effects also varied among different vegetation types. Generally, wood plants responded slower to SWC stress than forbs and the lag effect of SWC stress on woody plants lasted longer. Therefore, the effect of SWC stress is higher in forests and savannas, but lower in grasslands and open shrublands under SWC-stressed conditions (Supplementary Fig. S31a). Overall, atmospheric dryness is more related to vegetation changes on a short temporal scale, while SWC stress is effective in driving vegetation on a relatively long temporal scale in drylands.

Moreover, under atmospheric and soil dryness, SWC and vegetation growth maintain a nearly linear relationship, while the relationship between VPD and vegetation growth diverges more and more (Fig. 3b and c, and Supplementary Fig. S7h–k). Therefore, in the long term, SWC stress increasingly dominates vegetation growth.

The current debate about the driving forces of dryland ecosystems mainly stems from the differences in the objective variables, study area, data sources and methods used (Supplementary Table S1). First, previous assessments have mostly focused on a global or a specific regional scale, while targeting the largest drylands on our planet can decrease confounding effects of other non-drylands ecosystem processes. Second, we selected multi-source observation-based indices that measure different aspects of vegetation state, which has been proven to have higher accuracies than ecosystem process models. The relationships between water stress and vegetation growth are generally consistent for the NDVI, VOD and GPP data. However, they are not identical. Different aspects emphasized by each piece of data and their intrinsic features both can generate their distinct results. NDVI mainly reflects vegetation greenness and it also indicates LAI and chlorophyll content; VOD mainly represents vegetation water content; while GPP measures gross photosynthesis rates without necessarily transferring to vegetation growth considering plant respiration. Therefore, there must be differences in their findings regarding the estimated dryland ecosystems water-stressed state; however, the three independent data sources consistently support our inference of the enhanced dominance of SWC stress on the vegetation growth of Eurasian drylands.

## CONCLUSIONS

This study investigated the relative dominance of high VPD and low SWC on vegetation growth in Eurasian drylands. Our results indicated that SWC was a more influential stressor than VPD on vegetation growth over Eurasian drylands during 1982–2014, and the dominance of SWC stress is projected to further strengthen with future warming. Our findings can help to constrain model uncertainties in displaying water stress on ecosystems through the non-linear VPD–SWC–vegetation interactions.

For dryland ecosystems, future research could focus on detecting non-linearities or critical thresholds of their state changes and improving their representation in process-based models. More importantly, current temperatures are approaching or exceeding the optimal temperature for photosynthesis [60]. Research findings of this study are vital to reducing mortality risks due to water and heat stress in dryland ecosystems.

## METHODS

As shown in Table 1, a positive value of ΔNDVI (VPD${|}$SWC) or ΔNDVI (SWC${|}$VPD) means that vegetation growth is non-suppressed under atmospheric water stress (termed as ‘non-VPD-stressed’) or soil water stress conditions (termed as ‘non-SWC-stressed’), respectively. Accordingly, we defined those areas with positive ΔNDVI (VPD${|}$SWC) or ΔNDVI (SWC${|}$VPD) as ‘non-water-stressed ecosystems’. If ΔNDVI (VPD${|}$SWC) was positive, we defined the area as ‘non-VPD-stressed ecosystems’. Similarly, if ΔNDVI (SWC${|}$VPD) was positive, we defined the area as ‘non-SWC-stressed ecosystems’.

**Table 1.** The redefinitions of ecosystem water stress.

Variable name	Positive value	Negative value	Lower negative value between A and B
A: ΔNDVI (VPD${|}$SWC)	Non-VPD-stressed ecosystems	VPD-stressed ecosystems	Ecosystems dominated by VPD stress
B: ΔNDVI (SWC${|}$VPD)	Non-SWC-stressed ecosystems	SWC-stressed ecosystems	Ecosystems dominated by SWC stress

In contrast, a negative value of ΔNDVI (VPD${|}$SWC) or ΔNDVI (SWC${|}$VPD) means that vegetation growth is suppressed under atmospheric water stress (termed as ‘VPD-stressed’) or soil water stress conditions (termed as ‘SWC-stressed’), respectively. Accordingly, we defined those areas with negative ΔNDVI (VPD${|}$SWC) or ΔNDVI (SWC${|}$VPD) as ‘water-stressed ecosystems’. If ΔNDVI (VPD${|}$SWC) was negative, we defined the area as ‘VPD-stressed ecosystems’, and if ΔNDVI (SWC${|}$VPD) was negative, we defined the area as ‘SWC-stressed ecosystems’. For those areas with both negative ΔNDVI (VPD${|}$SWC) and ΔNDVI (SWC${|}$VPD) (i.e. compound water stress effects), the factor with a greater negative value was identified as the dominant water stress effect for vegetation growth. Similarly, the other three vegetation indices (i.e. VOD, GPP and LAI) were also used in accordance with the same definition.

## Supplementary Material

nwad108_Supplemental_File
