# Supplementary material for: Enhanced dominance of soil moisture stress on vegetation growth in Eurasian drylands
Source: Natl Sci Rev. 2023 Apr 24;10(8):nwad108. doi: 10.1093/nsr/nwad108 (PMC10306363; doi:10.1093/nsr/nwad108)
Supplement: nwad108_Supplemental_File [file nwad108_supplemental_file.pdf]

SUPPORTING INFORMATION

Enhanced dominance of soil moisture stress on vegetation growth in Eurasian drylands

Yu Zhang[1,2], Yangjian Zhang[1,2,*], Xu Lian[3,4], Zhoutao Zheng[1], Guang Zhao[1], Tao Zhang[5], Minjie Xu[5], Ke Huang[1,6], Ning Chen[7], Ji Li[8,1], Shilong Piao[3,9]

[1]Key Laboratory of Ecosystem Network Observation and Modeling, Institute of Geographic Sciences and Natural Resources Research, Chinese Academy of Sciences, Beijing 100101, China

[2]College of Resources and Environment, University of Chinese Academy of Sciences, Beijing 100190, China

[3]Sino-French Institute for Earth System Science, College of Urban and Environmental Sciences, Peking University, Beijing 100871, China

[4]Department of Earth and Environmental Engineering, Columbia University, New York 10027, USA

[5]College of Agronomy, Shenyang Agricultural University, Shenyang 110866, China

[6]Department of Geosciences and Natural Resource Management, University of Copenhagen, Copenhagen 1350, Denmark

[7]Key Laboratory of Wetland Ecology and Environment, Northeast Institute of Geography and Agroecology, Chinese Academy of Sciences, Changchun 130102, China

[8]Department of Geography, School of Geography and Information Engineering, China University of Geosciences, Wuhan 430078, China

[9]State Key Laboratory of Tibetan Plateau Earth System, Resources and Environment, Institute of Tibetan Plateau Research, Chinese Academy of Sciences, Beijing 100085, China

***Corresponding author.** E-mail: zhangyj@igsnrr.ac.cn

Supplementary Methods

Vegetation growth indices

Three satellite-observed vegetation indices as a proxy for vegetation condition were used in this study, including the third-generation biweekly Advanced Very High-Resolution Radiometer (AVHRR) normalized difference vegetation index (NDVI) (GIMMIS-NDVI3g) [1,2], the daily Ku-band vegetation optical depth (VOD) [3], and gross primary production (GPP) dataset based on NIRv (GPP_{NIRv}) [4]. We used three independent remote sensing datasets because each has its own limitations. For instance, NDVI is affected by soil background; VOD is influenced by soil moisture; and GPP depends on meteorological factors, furthermore, the fitness between NIRv and GPP has large uncertainty. All vegetation data were resampled to a 1° spatial resolution (Supplementary Table 2). The bi-weekly GIMMIS-NDVI3g data was composited into monthly values by maximum value compositing method. We only used vegetation index values averaged over the growing season period (April to October) to avoid spurious vegetation changes caused by snow presence in dormant seasons [2,5]. Based on the data availability of vegetation growth and soil water content datasets, NDVI and GPP data were analyzed for 1982–2014, and VOD data for 1988–2014 (Supplementary Table 2). GIMMIS-NDVI3g was used in the main text. Vegetation types were defined following the International Geosphere-Biosphere Program based on Terra and Aqua combined Moderate Resolution Imaging Spectroradiometer (MODIS) Land Cover Type (MCD12Q1) Version 6 data [6] and they were also used to exclude non-vegetation areas.

Drought indices

Vapor pressure deficit (VPD) and soil water content (SWC) are the two common indices of plant water availability, reflecting atmospheric and soil dryness, respectively [7]. Four climatic data sets

were used to investigate the dynamics of VPD, including TerraClimate [8], the European Centre for Medium-Range Weather Forecasts (ECMWF) Reanalysis-Interim (ERA-Interim) [9], the Modern-Era Retrospective analysis for Research and Applications, Version 2 (MERRA-2) [10], and the ECMWF Reanalysis Version 5 for land applications (ERA5-Land)[11] (Supplementary Table 2). Two observation-driven soil moisture data sets were used to quantify the spatiotemporal dynamics of SWC. Since vegetation mainly absorbs water through the root system, the root zone soil moisture data as a proxy for total soil water content (SWC), was also used [12], which includes satellite-based soil moisture from GLEAMv3.5a [13–15] and the Global Land Data Assimilation System Version 2 (GLDAS-2) from the Noah Model 3.6 [16] (Supplementary Table 2). The two soil moisture products were generated from different data assimilation systems and different climatic forcing inputs. ERA-Interim VPD and GLEAM SWC were used.in the main text. All VPD and SWC data sets were resampled to a 1° spatial resolution. To ensure comparability among different observation-driven data sets, we transformed the average total soil moisture of the study domain (Supplementary Fig. 1) as a fraction (%) of the simulated climatological mean during the baseline period 1961–1990 [17].

Aridity index

The aridity index (AI) measures the balance between the atmospheric water supply to the land (precipitation) and its demand from the land surface (potential evaporation, Ep). To consolidate the study findings and avoid bias from data uses, we used five precipitation products and three reanalysis-based Ep products (Supplementary Table 2), to estimate the AI and delineate drylands extent. We derived AI estimates by combining all possible pairs of precipitation and Ep data-based products. For the longer 1958–2014 period, we derived nine ensemble members of AI using three

precipitation products (the Climatic Research Unit TS4.05 (CRUTS4.05) [18], the Princeton Global Meteorological Forcing v3 (PGFv3) [19], and the University of Delaware v5.01 (Udelv5.01) [20]) and three Ep products (GLDAS-2 [16], the National Center for Environmental Prediction/the National Center for Atmospheric Research (NCEP/NCAR) [21] and PGFv3 [19]). For the shorter 1982–2014 period, we derived six members of AI using all available precipitation and Ep databases. All AI data sets were aggregated to a 1° spatial resolution. The reliabilities of the 15 members were verified by comparing each annual mean AI (Supplementary Fig. 2).

Dynamic global vegetation models

We used 18 GPP and 14 leaf area index (LAI) simulations of offline dynamic global vegetation models (DGVMs) from the "Trends in net land atmosphere carbon exchange" (TRENDY-v9) project for 1982–2014 (Supplementary Table 3). For TRENDY DGVMs, we used S3 simulations that incorporated changes in climate forcing, rising atmospheric CO_2 concentrations, and land-use change. GPP and LAI for the vegetated land were resampled to a 1° spatial resolution, and aggregated to the growing season period (April to October) on a yearly timescale.

Earth system model outputs

We used monthly outputs of GPP, near-surface air temperature (T_{air}), near-surface relative humidity (RH), and total soil water content (SWC) from eleven Earth system models (ESMs) (Supplementary Table 4) that participated in simulations of the Coupled Model Intercomparison Project Phase 6 (CMIP6) under Shared Socioeconomic Pathways (SSPs) 3-7.0 and 5-8.5 scenarios for 2015–2100 (https://esgf-node.ipsl.upmc.fr/search/cmip6-ipsl/). Variables for the vegetated land were resampled to a 1° spatial resolution, and aggregated to the growing season period (April to October) on a yearly timescale.

Flux and environmental measurements

The carbon flux monitoring was carried out at Naqv alpine meadow ecosystem (AME) station (31.64°N, 92.01°E, 4600 m above sea level), which is a standard observation site of the China Flux Observation and Research Network. The station has a short growing season due to low temperatures, low SWC, and concentrated precipitation in Summer [22]. At this study site, Kobresia pygmaea accounts for about 70% of the total community aboveground biomass and 90% of the total community coverage. Thus, we used the phenological period of Kobresia Pygmaea to represent the growing season length (GSL) in AME. We randomly selected ten clusters of Kobresia Pygmaea in a circular area with a radius of 200 m around the flux tower, marked them with flags and observed them daily. We recorded the greening time of each cluster and calculated the average value as DOY at the start of the growing season. We recorded the total yellowing time of each individual plant and calculated the average value as DOY at the end of the growing season. For more details about flux data processing and phenological observation, see the Ref. [22].

Drylands extent with different aridity metrics

As a commonly used land surface aridity index, AI possesses high uncertainty in fully representing the complexity of aridity changes [17]. Meteorological conditions shape the geographic patterns of VPD and SWC at the land surface. We characterized how these aridity metrics had changed in recent decades (1958–2014; 1982–2014) as follows.

We first established an empirical relationship between a given aridity metric (VPD and SWC) and AI using all grid points in the AI-defined drylands (corresponding to AI = 0.65; Supplementary Fig. 1) [17,23–25]. Considering potential non-linearity in the relationship, we

tested three different empirical models: linear, polynomial, and exponential. The model with the maximum R^2 was adopted to describe the relationship between a given variable and AI [17]. With the pre-established regression models, we then calculated the respective thresholds of VPD and SWC for delineating dryland extent (Supplementary Fig. 4), based on which year-to-year variations of drylands were displayed. The baseline period was set as 1961–1990 (or a subset of years thereof depending on data availability) [17]. The fraction indices of f_{atm} and f_{soil}, for VPD and SWC, respectively, represent percentage changes relative to the baseline, where a positive (negative) value implies an expansion (contraction) of drylands compared to the baseline period of 1961–1990.

Percentile binning

To investigate yearly vegetation response to water stress, the binning method was applied [7]. This method is capable of decoupling VPD and SWC changes, describing potential non-linearity, and observing correlated and causal relationships. For each pixel, we determined segmentation threshold values of the 20th, 40th, 60th, 80th, and 100th percentile of VPD and SWC, which will later be used to bin the data. Data of all variables (VPD, SWC, and vegetation growth indices) are sorted into 5 bins according to the 0–20th, 20–40th, 40–60th, 60–80th, and 80–100th percentiles of VPD or SWC. Next, within each SWC bin (f = 1, 2, 3, 4, 5), the ranking from minimum $n_{f,\min}$ to maximum $n_{f,\max}$ was determined based on the f th VPD bin and predetermined VPD threshold value. Similarly, in each VPD bin (g = 1, 2, 3, 4, 5), the ranking from minimum $m_{g,min}$ to maximum $m_{g,max}$ ranking was determined by the g th SWC bin and the threshold value. The binned averages were used to quantify the stresses of SWC and VPD on vegetation growth indices (i.e., NDVI, VOD, GPP, and LAI).

For example, VPD stress on NDVI after excluding the effects of VPD-SWC coupling (termed ΔNDVI (VPD|SWC)) was derived from the changes in NDVI from low VPD to high VPD at each SWC bin. We calculated the difference between the highest VPD and the lowest VPD corresponding to NDVI in each SWC bin (ΔNDVI (VPD|SWC)):

$$\Delta\mathrm{NDVI(VPD|SWC)} = \frac{1}{F}\sum_{f=1}^{F}\mathrm{NDVI}_{f,n_{f,\max}} - \mathrm{NDVI}_{f,n_{f,\min}}$$

where F is the total number of SWC bins, f is the specific SWC bin number, $n_{f,\max}$ and $n_{f,\min}$ is the maximum and minimum VPD bin number at SWC bin f.

In the same way, SWC stress on NDVI without VPD-SWC coupling (termed ΔNDVI (SWC|VPD)) was derived from the changes in NDVI from high SWC to low SWC at each VPD bin. We calculated the difference between the lowest SWC and the highest SWC corresponding to NDVI in each VPD binning (ΔNDVI (SWC|VPD)):

$$\Delta\mathrm{NDVI(SWC|VPD)} = \frac{1}{G}\sum_{g=1}^{G}\mathrm{NDVI}_{m_{g,\min},g} - \mathrm{NDVI}_{m_{g,\max},g}$$

where G is the total number of VPD bins, g is the specific VPD bin number, $m_{g,\min}$ and $m_{g,\max}$ is the minimum and maximum SWC bin number at the VPD bin g .

In the Liu et al.' (2020)[7] paper, the percentile bins were conducted using daily data because VPD and soil moisture are diagnosed to be decoupled at the daily or finer scales [7]. To further test the long-term response of vegetation to water stress, in addition to the logical function based on prior knowledge, we also examined the Spearman partial correlation between vegetation indices and SWC or VPD after statistically controlling changes in the other drivers. For example, the partial correlation coefficient between NDVI and SWC was calculated by excluding the influence of VPD.

Supplementary Figures

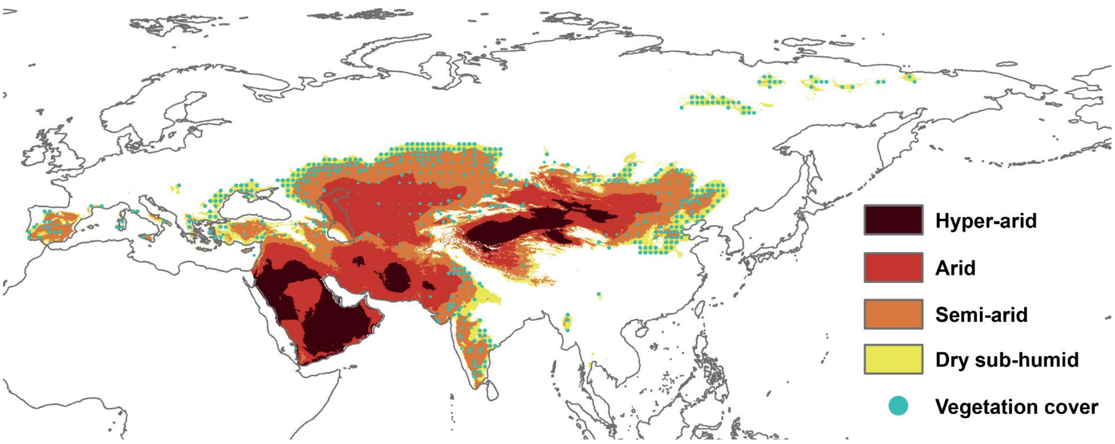

Supplementary Figure 1. The extent and types of aridity-index-defined drylands over Eurasia for 1961–1990, based on the TerraClimate dataset with high spatial resolution [8]. The cyan dots illustrate vegetation cover for 1982–1990, with larger dots indicating denser vegetation cover.

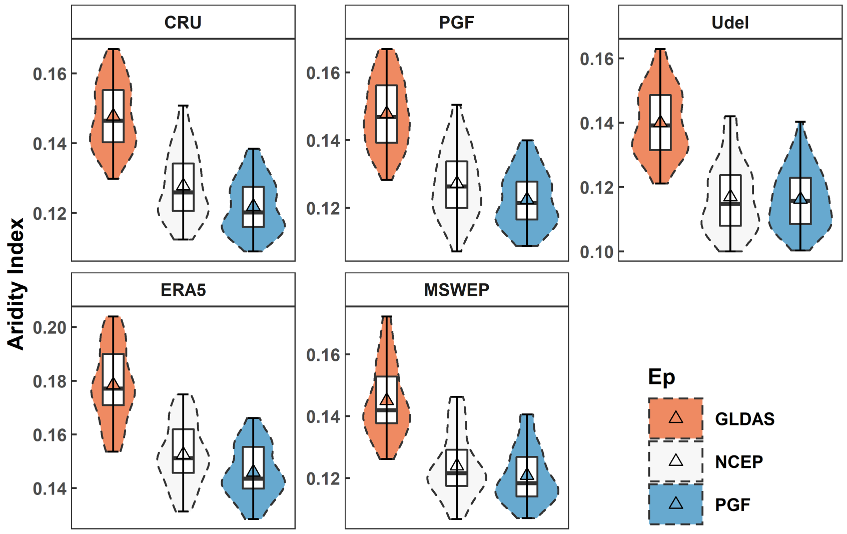

Supplementary Figure 2. Aridity index (AI) changes over Eurasian drylands evaluated by 15 different ensemble members. Nine ensemble members of aridity index (AI) for the longer 1958–2014 period, using three precipitation products (CRU, PGF, and Udel) and three potential evapotranspiration (Ep) products (GLDAS, NCEP and PGF) were derived. For the shorter 1982–2014 period, six ensemble members of AI were derived.

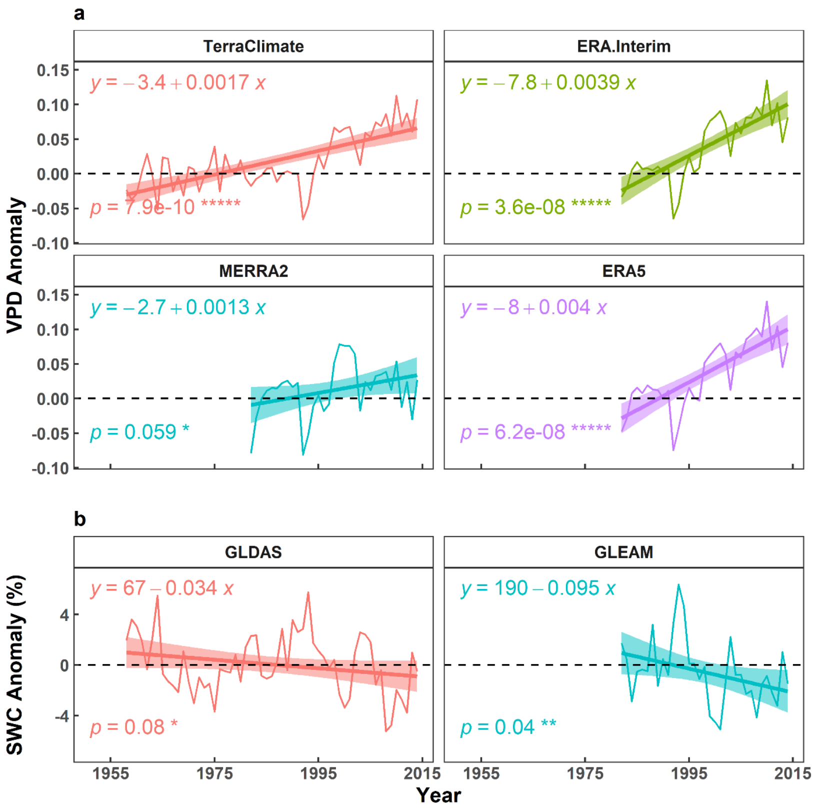

Supplementary Figure 3. Anomalies of annual mean vapor pressure deficit (VPD) and soil water content (SWC) changes from various data sources over Eurasian drylands. **a–b** Anomalies of VPD (**a**) and SWC (**b**), averaged over aridity index-defined baseline regions of drylands for 1961–1990. The shaded areas represent the 95% confidence intervals of each dataset. Statistical significances are shown as symbols '*****', '**', and '*' denoting $p < 0.001$, $p < 0.05$, and $p < 0.1$, respectively. Anomalies are calculated by subtracting the climatological mean of 1961–1990 (or a subset of years within this period, depending on the temporal coverage of data).

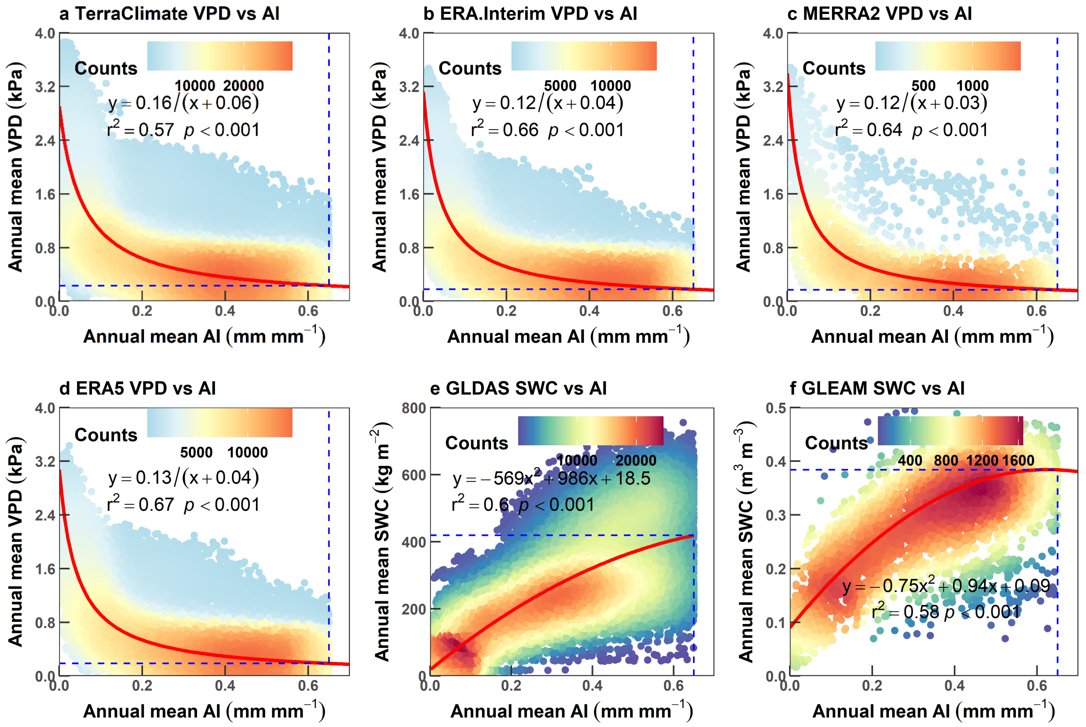

Supplementary Figure 4. Relationship between aridity index (AI) and other aridity metrics over Eurasian drylands. The best-fitted relationships between AI and other aridity metrics were established in a similar way as the Ref. [17]. Each panel shows the relationship between a specific aridity measure from different data sources and AI from an average of precipitation and potential evapotranspiration (Ep) data combinations. The VPD and SWC denote vapor pressure deficit and soil water content, respectively.

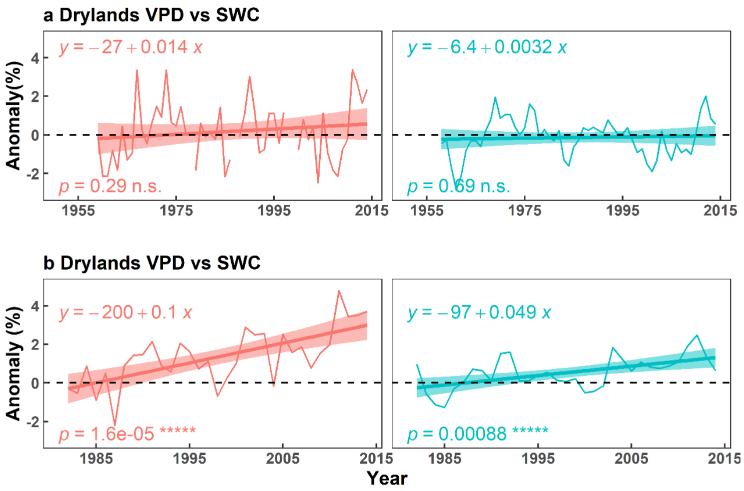

Supplementary Figure 5. Decoupling of vapor pressure deficit (VPD, red line) and soil water content (SWC, cyan line) over Eurasian drylands during 1958–2014 (**a**) and 1982–2014 (**b**). Similar to regions with AI < 0.65, f_{atm} and f_{soil} are computed using threshold values of the corresponding metric. Anomalies are computed by subtracting the climatological mean of 1961–1990 (or a subset of years during this period, depending on the temporal coverage of data). The shaded areas represent the 95% confidence intervals. Statistical significances are shown as symbols '*****' and 'n.s.', denoting $p < 0.001$ and $p > 0.1$, respectively. The VPD and SWC were calculated as the average of multiple data, respectively.

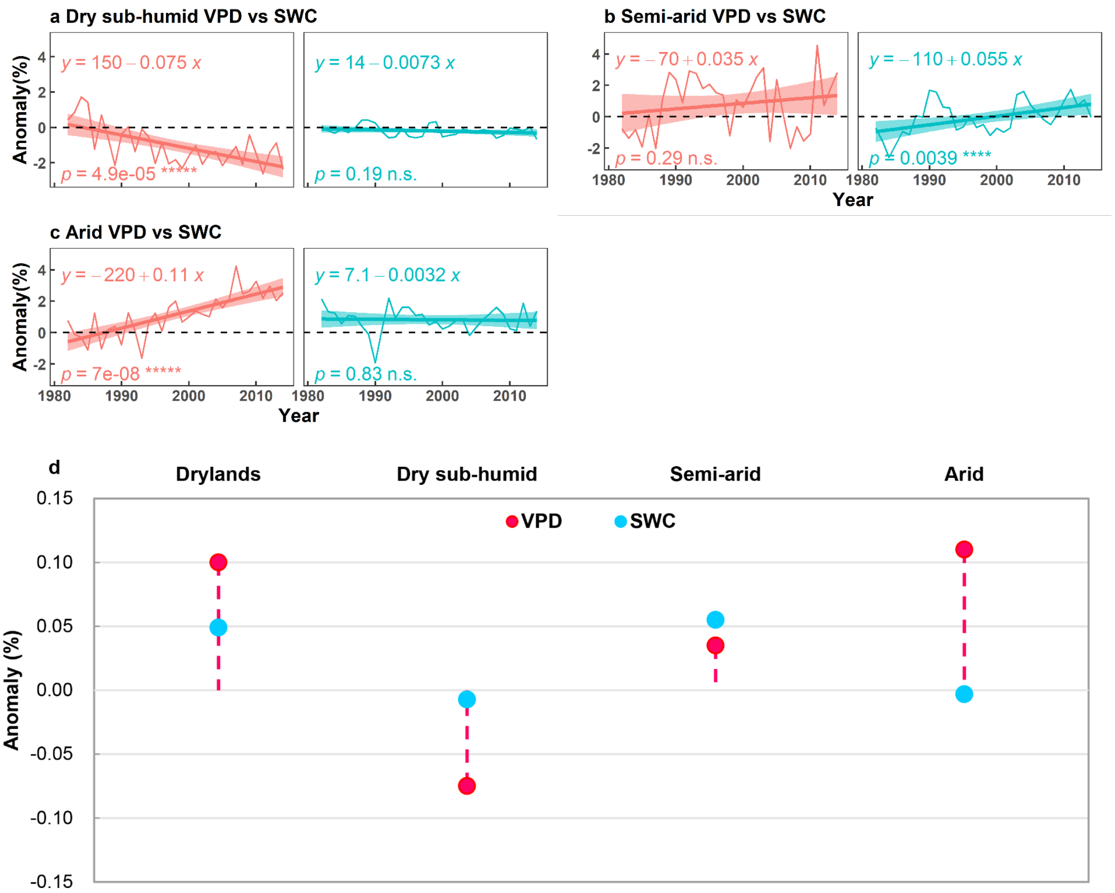

Supplementary Figure 6. Decoupling of vapor pressure deficit (VPD, red line) and soil water content (SWC, cyan line) during 1982–2014. **a–c** Anomalies (as %) of area fraction of drylands evaluated by VPD (f_{atm}) and SWC (f_{soil}) during 1982–2014 for dry sub-humid (**a**), semi-arid (**b**), and arid (**c**) regions. **d** Trends magnitudes of f_{atm} and f_{soil} in aridity gradients. Similar to regions with aridity index (AI) < 0.65, f_{atm} and f_{soil} are computed using threshold values of the corresponding metric. Anomalies are computed by subtracting the climatological mean of 1961–1990 (or a subset of years during this period, 1982–1990). The shaded areas represent the 95% confidence intervals. Statistical significances are shown as symbols '*****', '****', and 'n.s.', denoting $p < 0.001$, $p < 0.005$ and $p > 0.1$, respectively. The VPD and SWC values were calculated as the average of multiple data, respectively.

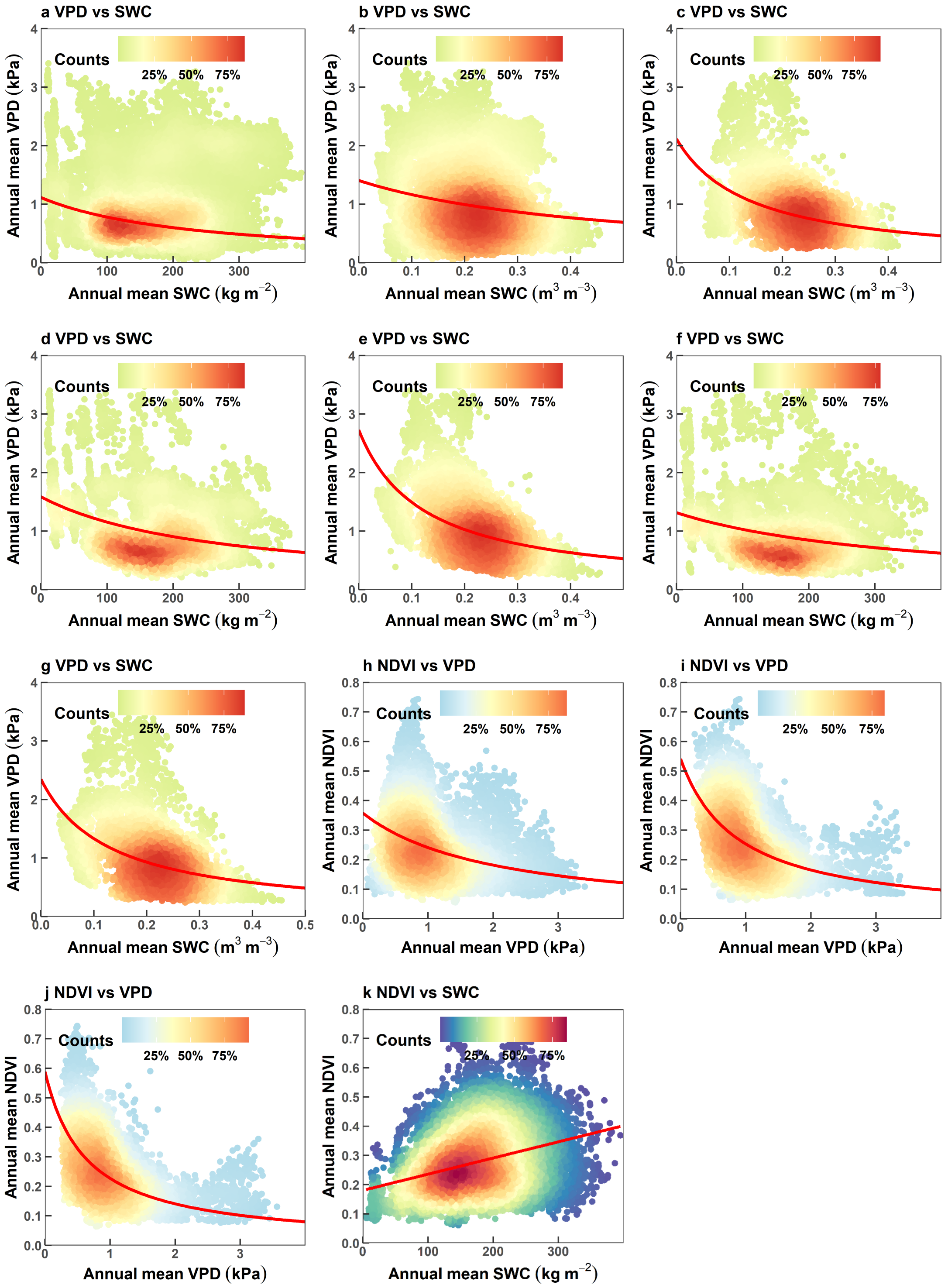

Supplementary Figure 7. Same as Fig. 3, but using TerraClimate VPD vs GLDAS SWC (**a**), TerraClimate VPD vs GLEAM SWC (**b**), ERA-Interim VPD vs GLDAS SWC (**c**), MERRA2 VPD vs GLDAS SWC (**d**), MERRA2 VPD vs GLEAM SWC (**e**), ERA5-Land VPD vs GLDAS SWC (**f**), ERA5-Land VPD vs GLEAM SWC (**g**),

GIMMIS NDVI vs TerraClimate VPD (**h**), GIMMIS NDVI vs MERRA2 VPD (**i**), GIMMIS NDVI vs ERA5-Land VPD (**j**), and GIMMIS NDVI vs GLDAS SWC (**k**).

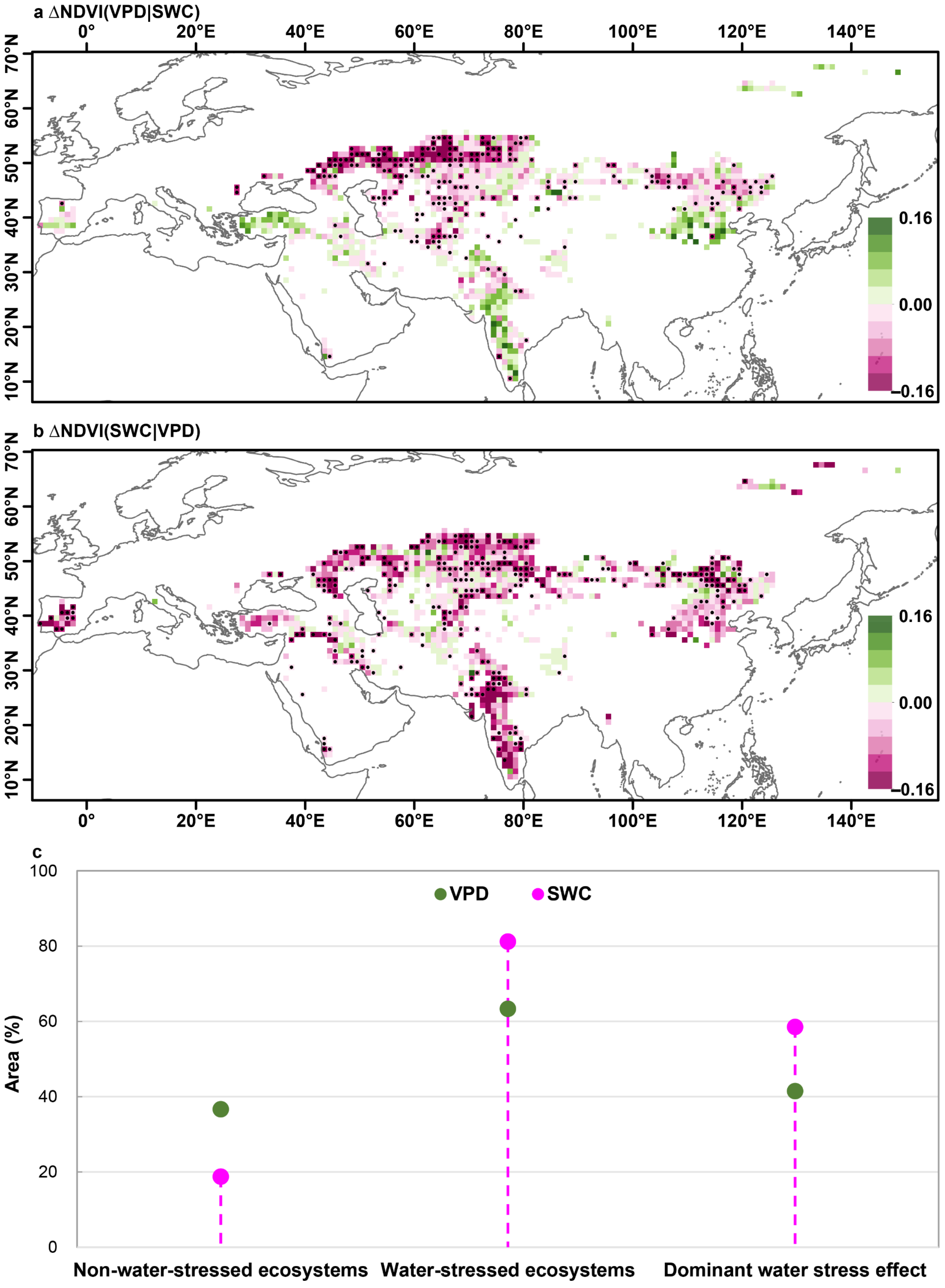

Supplementary Figure 8. Same as Fig. 4, but using TerraClimate VPD and GLDAS SWC.

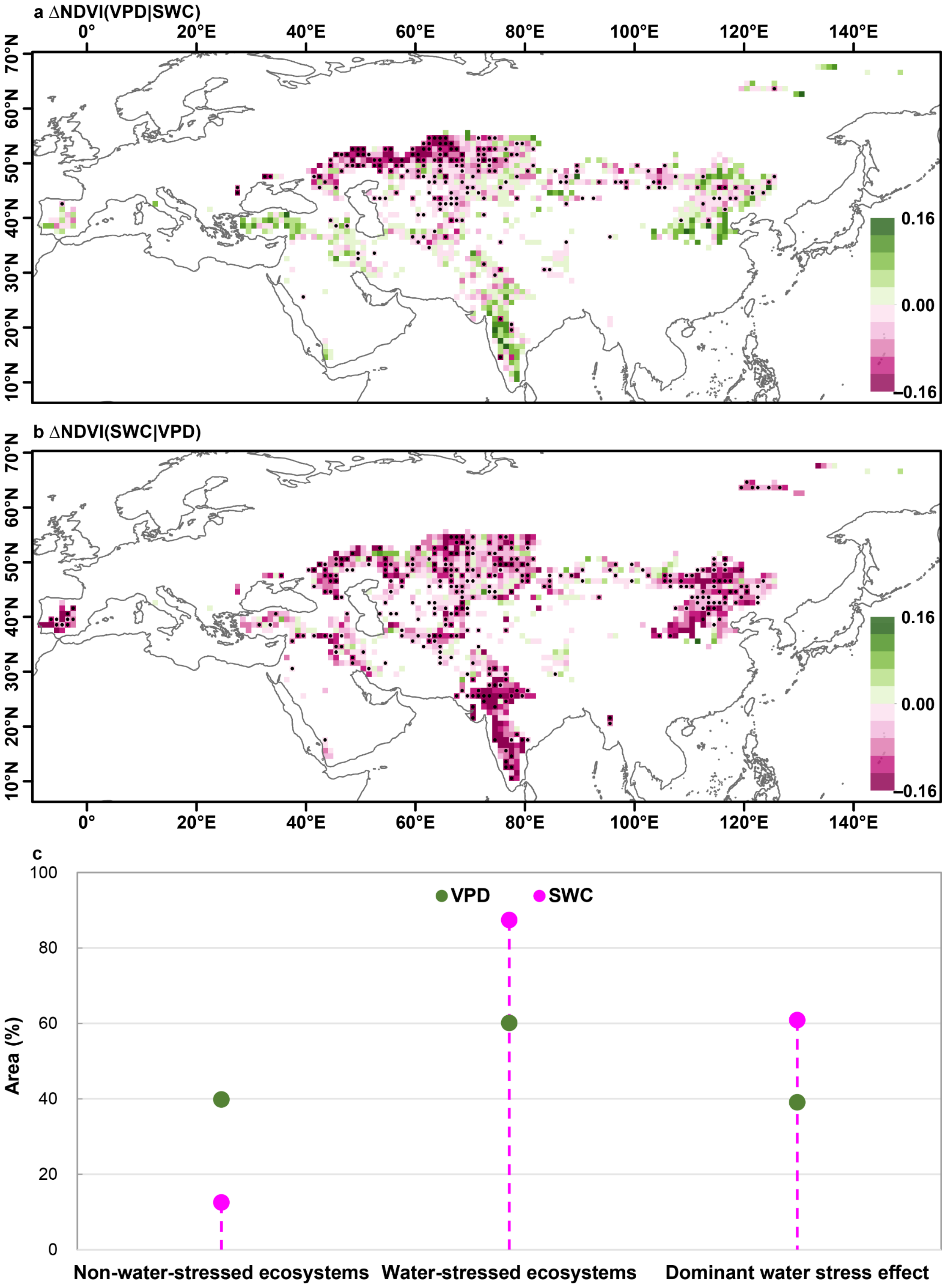

Supplementary Figure 9. Same as Fig. 4, but using TerraClimate VPD and GLEAM SWC.

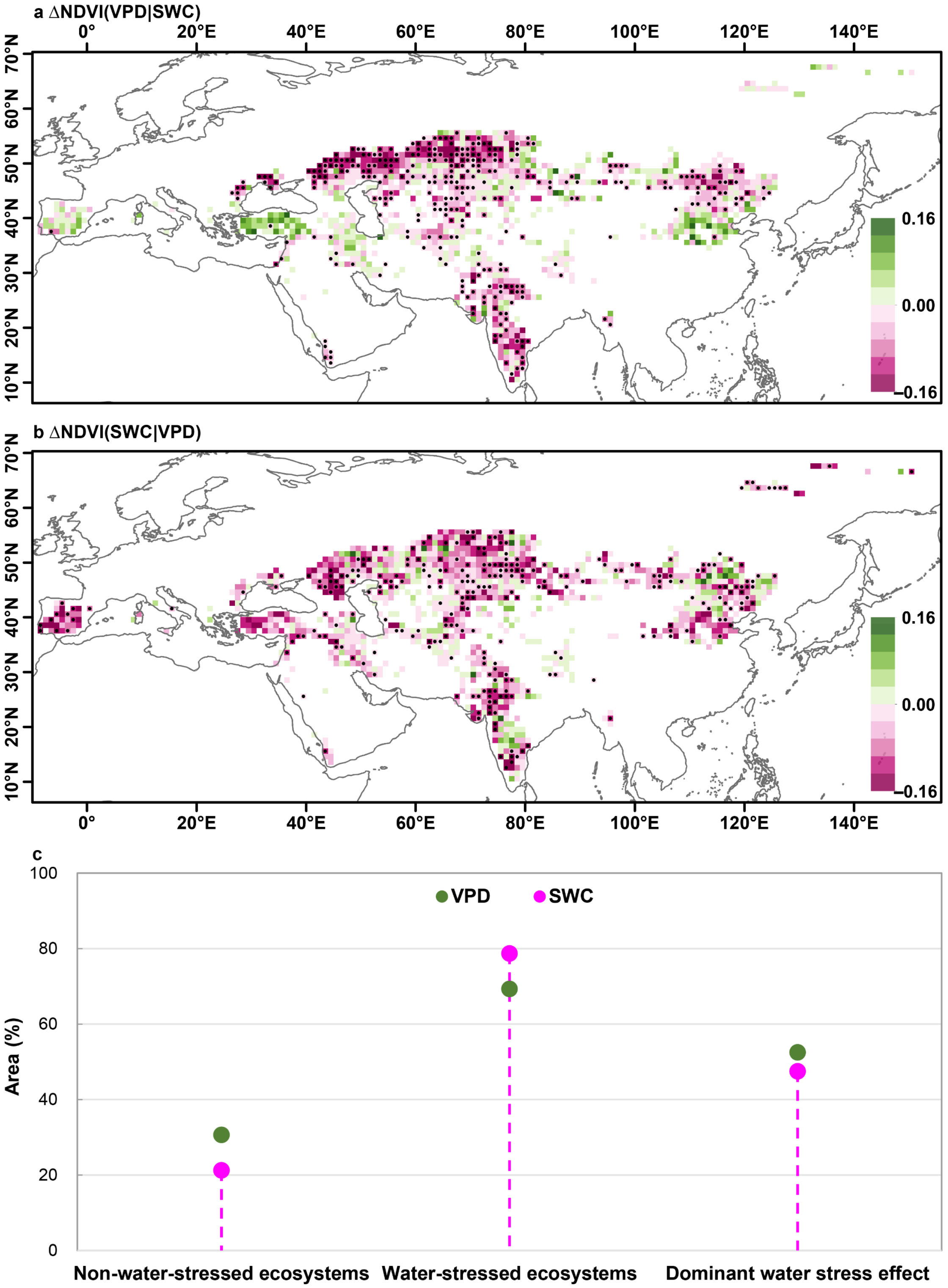

Supplementary Figure 10. Same as Fig. 4, but using ERA-Interim VPD and GLDAS SWC.

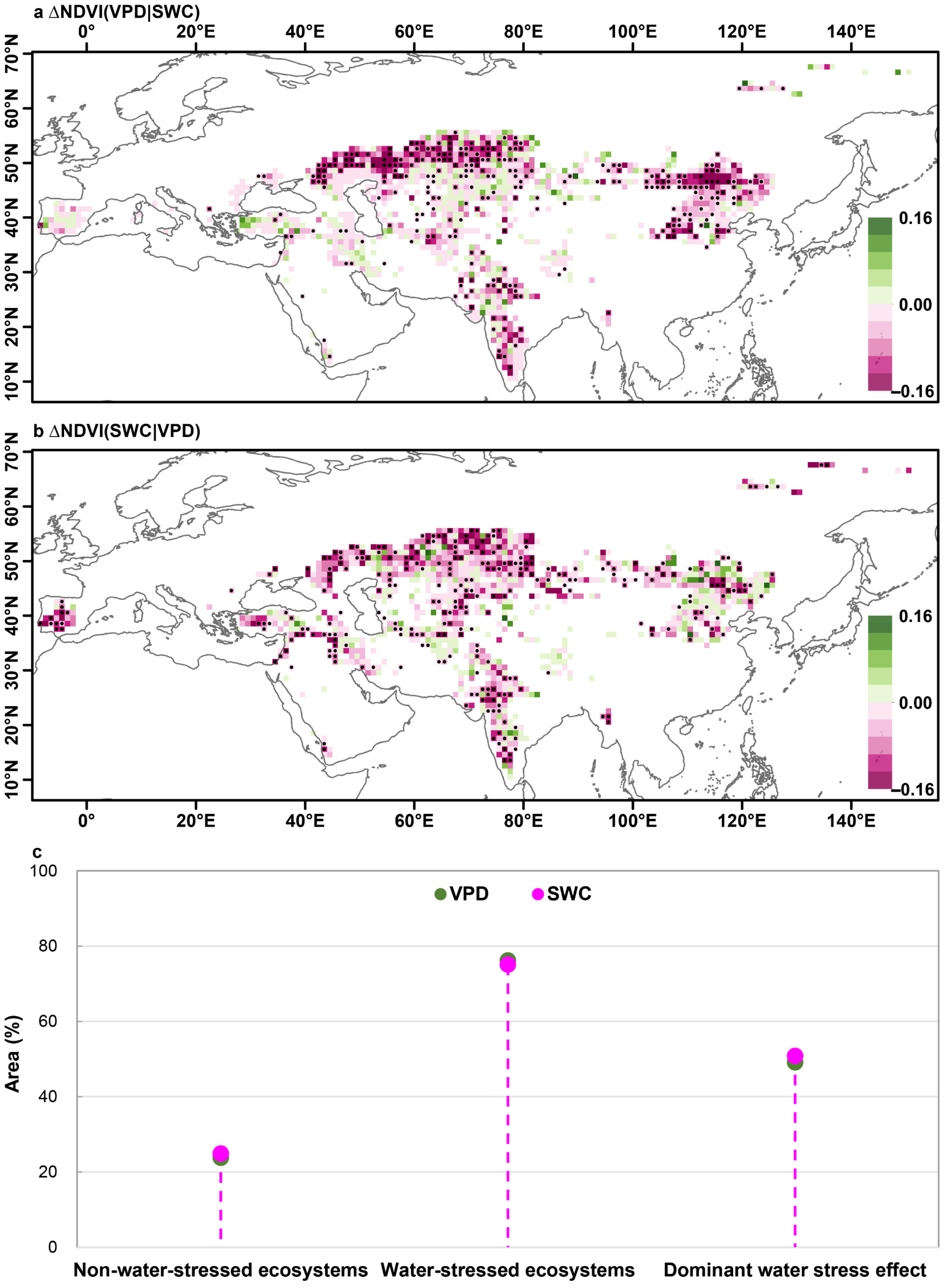

Supplementary Figure 11. Same as Fig. 4, but using MERRA2 VPD and GLDAS SWC.

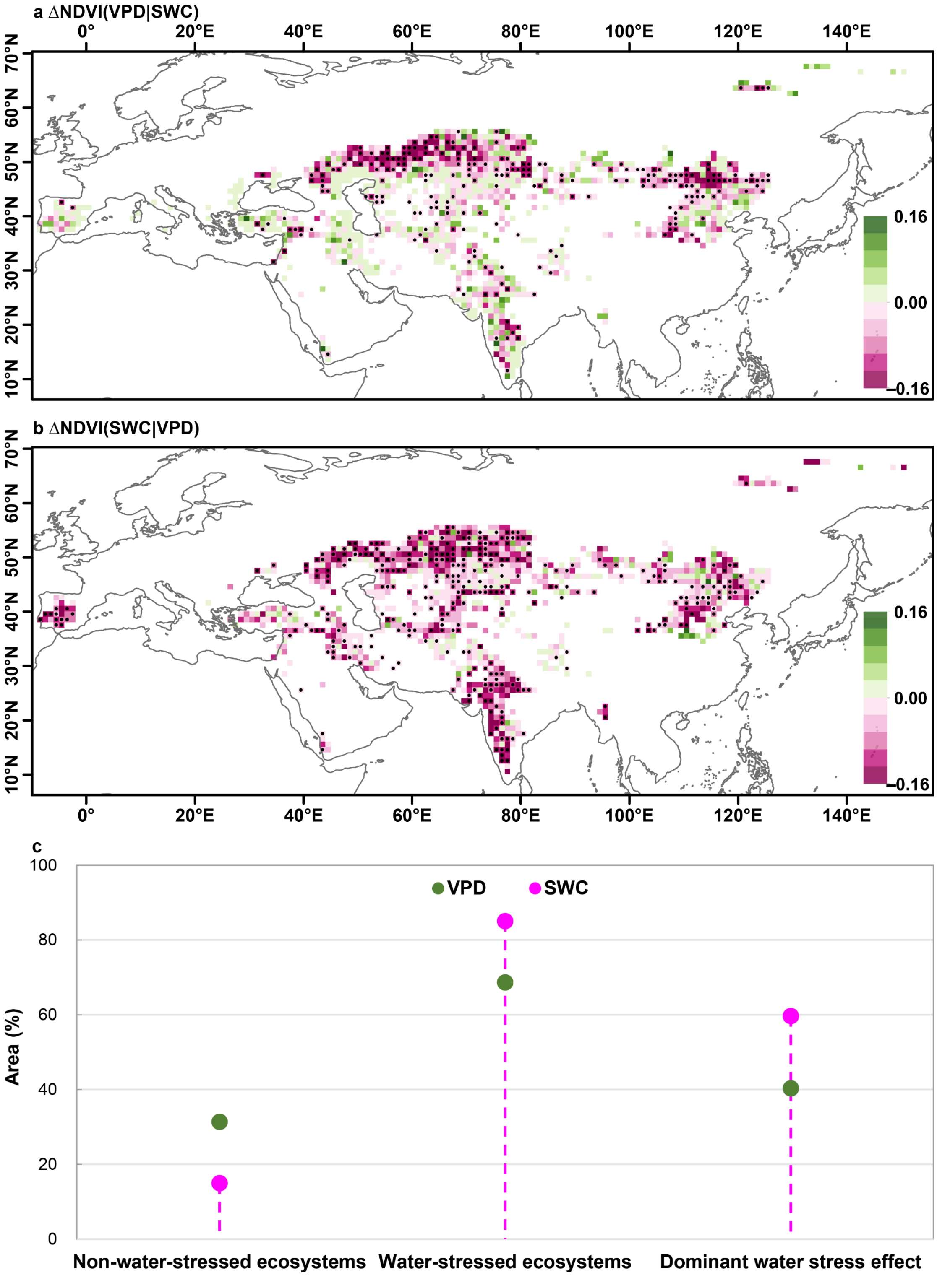

Supplementary Figure 12. Same as Fig. 4, but using MERRA2 VPD and GLEAM SWC.

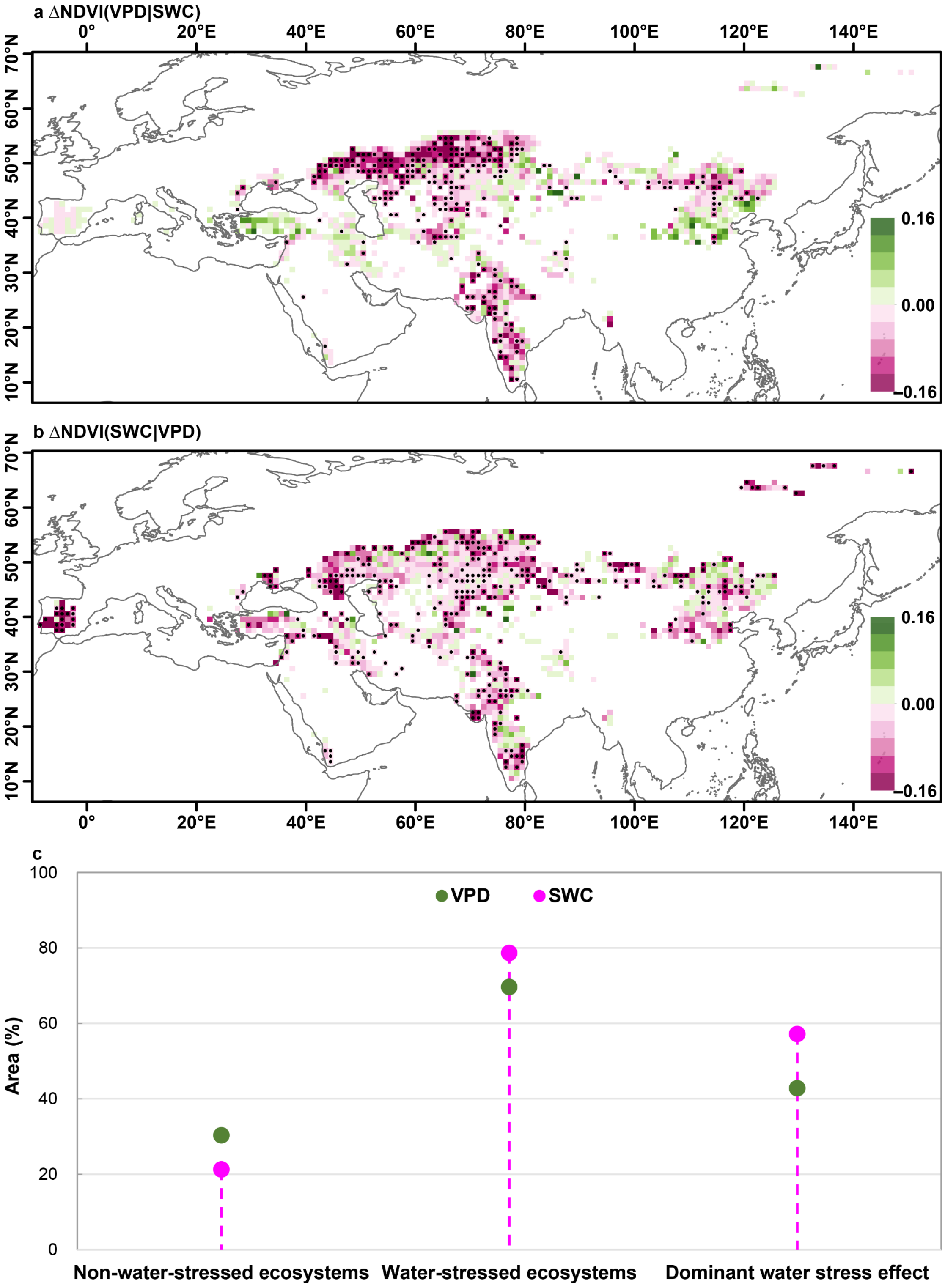

Supplementary Figure 13. Same as Fig. 4, but using ERA5-Land VPD and GLDAS SWC.

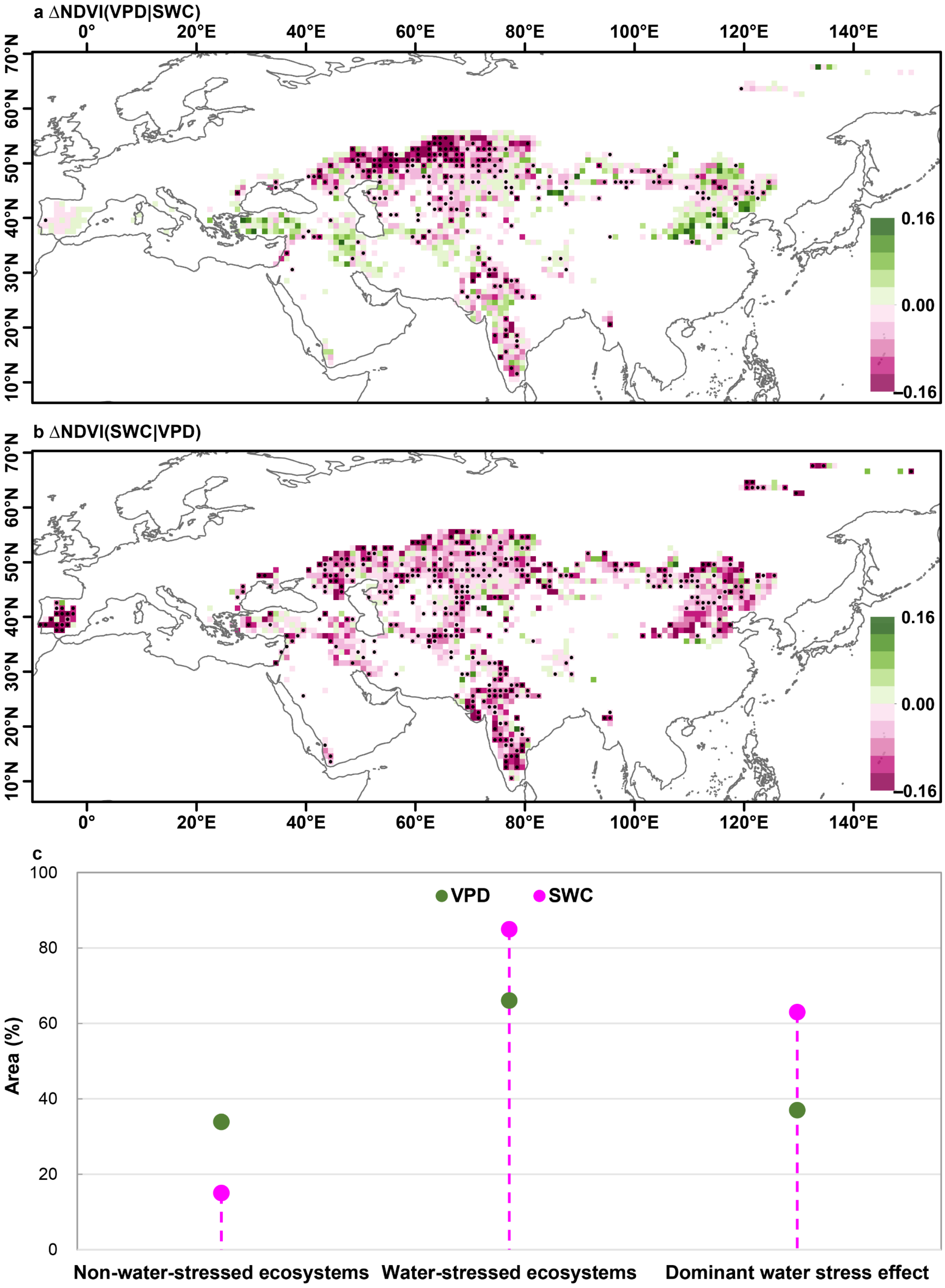

Supplementary Figure 14. Same as Fig. 4, but using ERA5-Land VPD and GLEAM SWC.

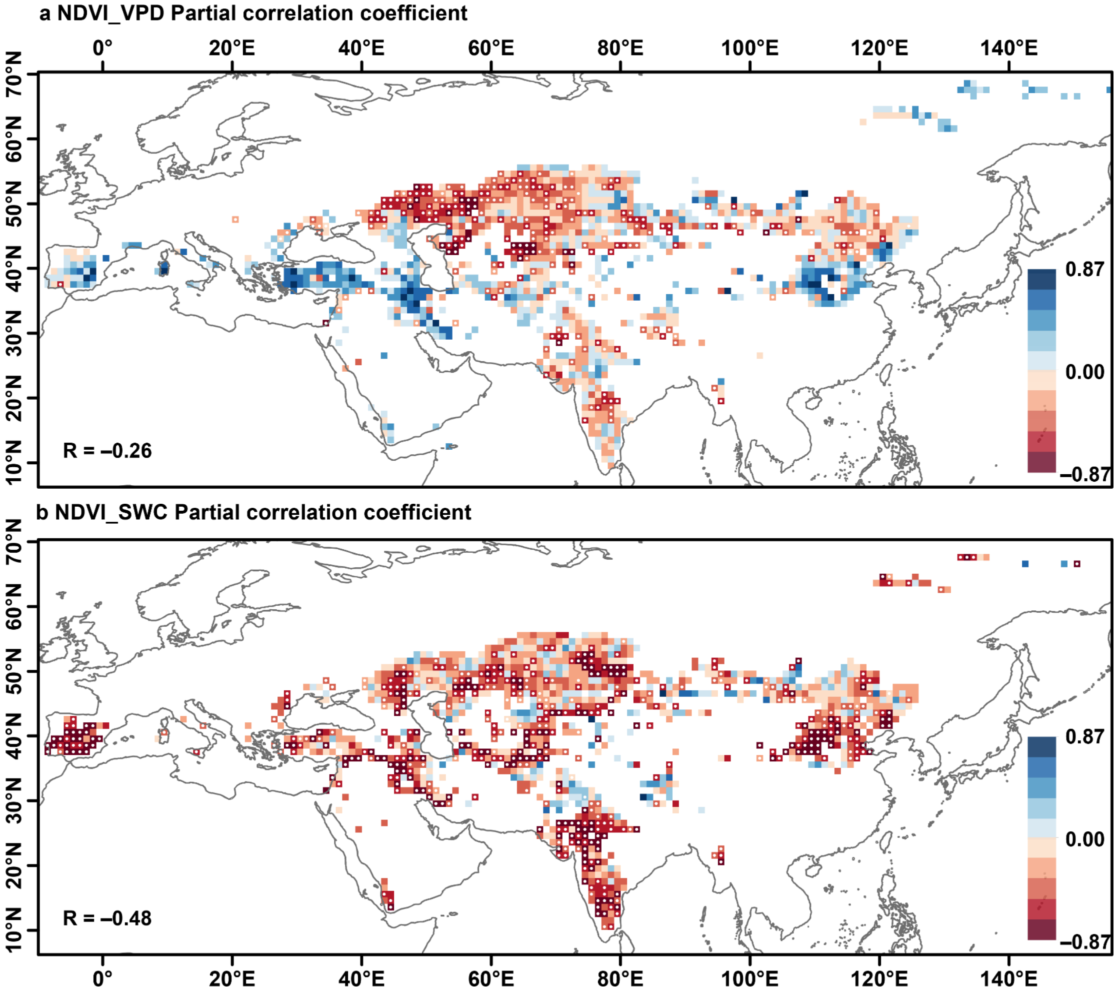

Supplementary Figure 15. Spatial patterns of the partial correlation coefficient between normalized difference vegetation index (NDVI) and vapor pressure deficit (VPD) (**a**) (or soil water content (SWC) (**b**)) over Eurasian drylands from 1982 to 2014. White circles indicate significant Spearman correlations with $p < 0.05$. Considering the opposite sign between SWC and VPD, the partial correlation between NDVI and SWC is displayed in number opposite to the actual value. ERA-Interim VPD, GLEAM SWC, and GIMMIS NDVI were used.

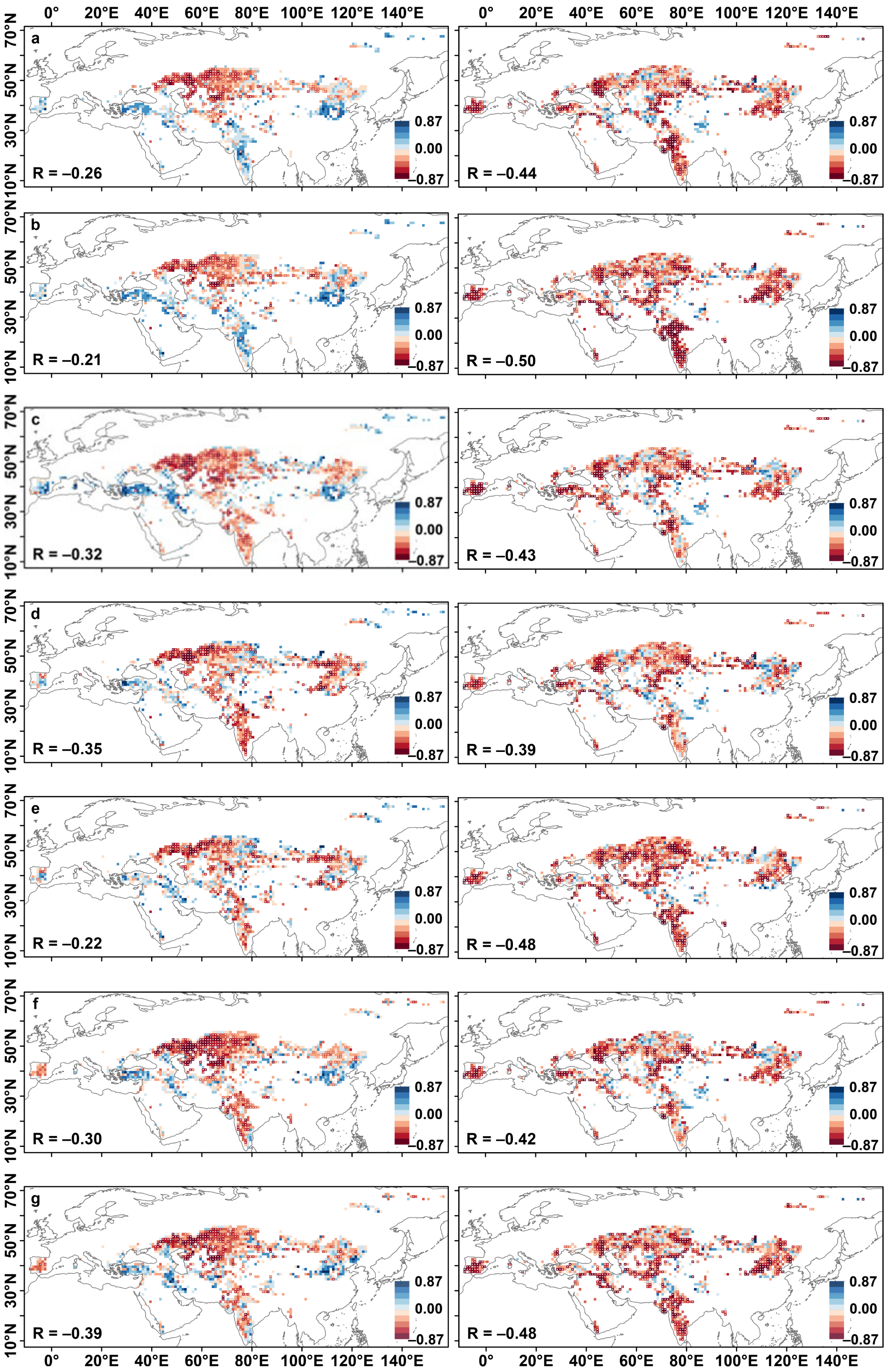

Supplementary Figure 16. Spatial patterns of the partial correlation coefficient between normalized difference vegetation index (NDVI) and vapor pressure deficit (VPD) (left panels) (or soil water content (SWC) (right

panels)) over Eurasian drylands from 1982 to 2014, based on TerraClimate VPD and GLDAS SWC (**a**); TerraClimate VPD and GLEAM SWC (**b**); ERA-Interim VPD and GLDAS SWC (**c**); MERRA2 VPD and GLDAS SWC (**d**); MERRA2 VPD and GLEAM SWC (**e**); ERA5-Land VPD and GLDAS SWC (**f**); ERA5-Land VPD and GLEAM SWC (**g**). White circles indicate significant Spearman correlations with $p < 0.05$. Considering the opposite sign between SWC and VPD, the partial correlation between NDVI and SWC is displayed in number opposite to the actual value.

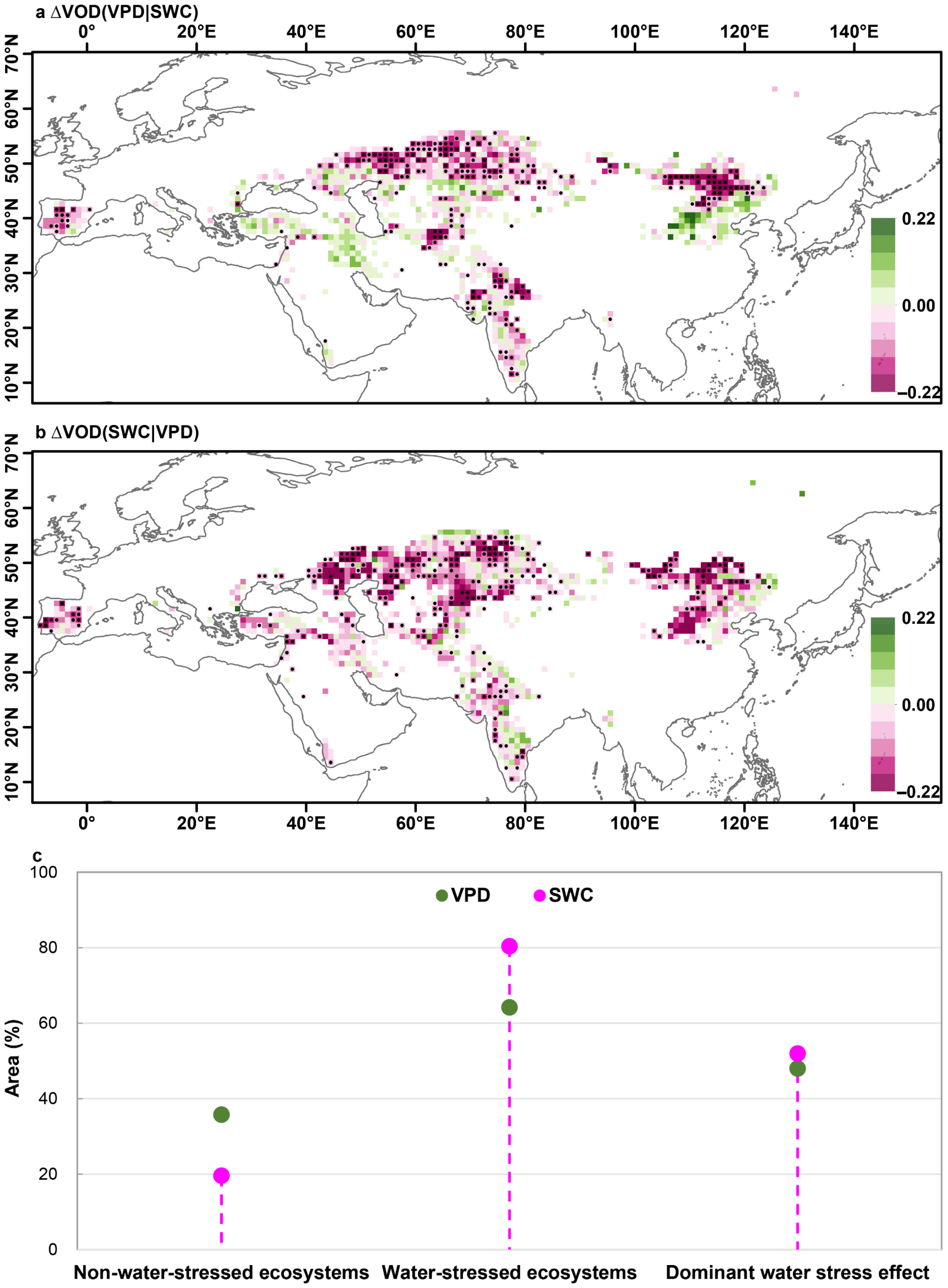

Supplementary Figure 17. Relative roles of vapor pressure deficit (VPD) and soil water content (SWC) on vegetation optical depth (VOD) over Eurasian drylands during 1988–2014. We used an ensemble mean of four VPD products, as well as two products for SWC to estimate. **a–b** the changes in VOD caused by high VPD (**a**) and low SWC (**b**), and their dominant water stress effect (black circles; see Methods: Water-stressed ecosystems

redefinitions). **c** Relative contributions of high VPD and low SWC to the variation in VOD.

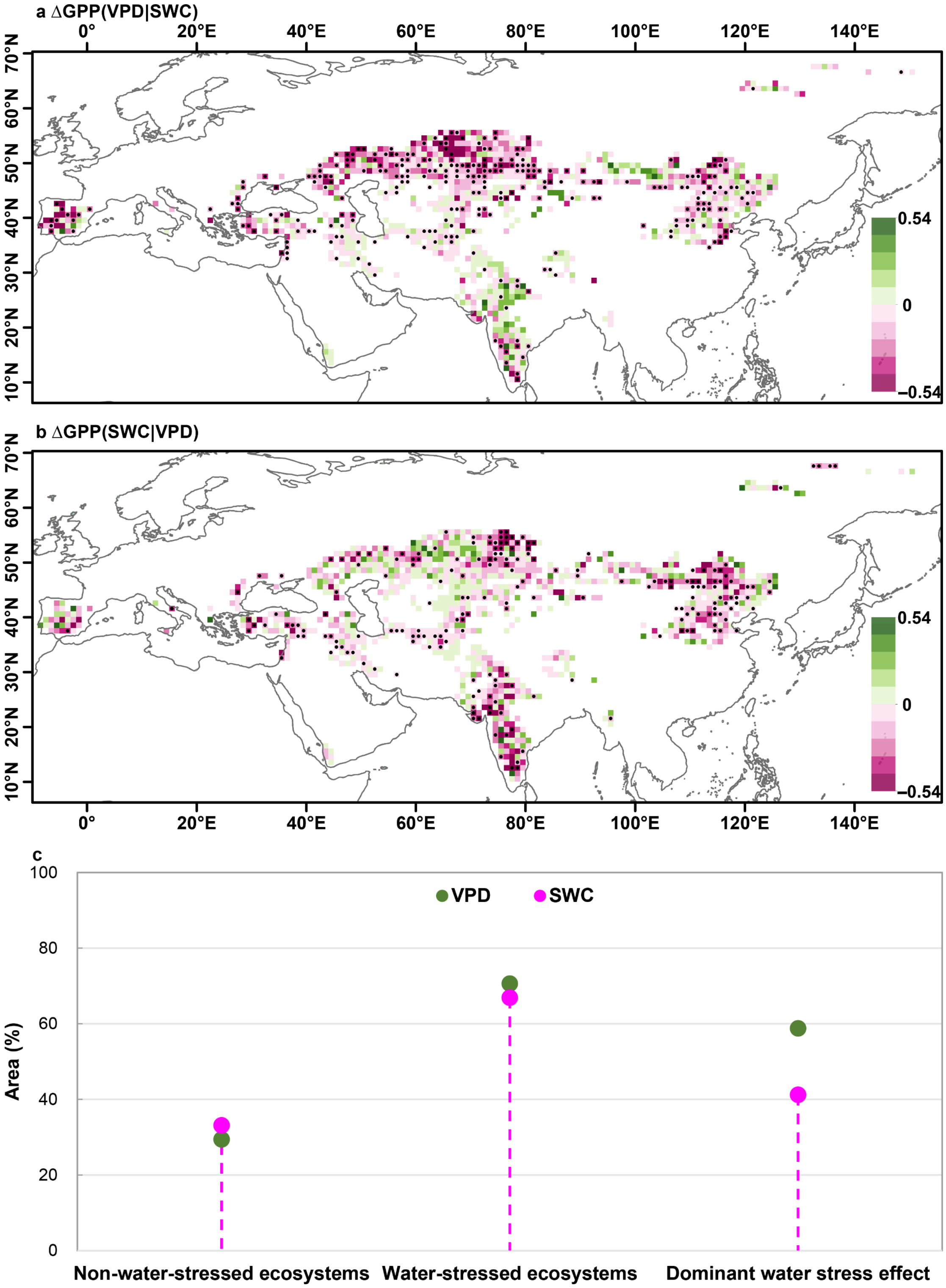

Supplementary Figure 18. Relative roles of vapor pressure deficit (VPD) and soil water content (SWC) on gross primary production (GPP) over Eurasian drylands for 1982–2014. We used an ensemble mean of four VPD products, as well as two products for SWC to estimate. **a–b** the changes in GPP caused by high VPD (**a**) and low

SWC (**b**), and their dominant water stress effect (black circles; see Methods: Water-stressed ecosystems redefinitions). **c** Relative contributions of high VPD and low SWC to the variation in GPP.

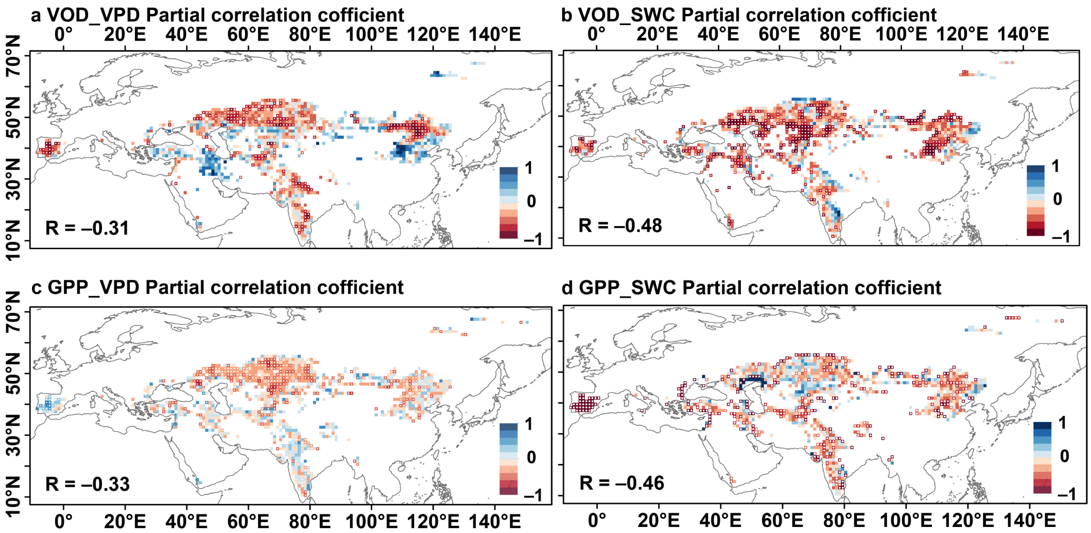

Supplementary Figure 19. Spatial patterns of the partial correlation coefficient between vegetation optical depth (VOD) (or gross primary production (GPP)) and vapor pressure deficit (VPD) (**a, c**) (or soil water content (SWC) (**b, d**)) over Eurasian drylands. White circles indicate significant Spearman correlations with $p < 0.05$. Considering the opposite sign between SWC and VPD, the partial correlation between VOD (or GPP) and SWC is displayed in number opposite to the actual value. The VPD and SWC values were calculated as the average of multiple data, respectively.

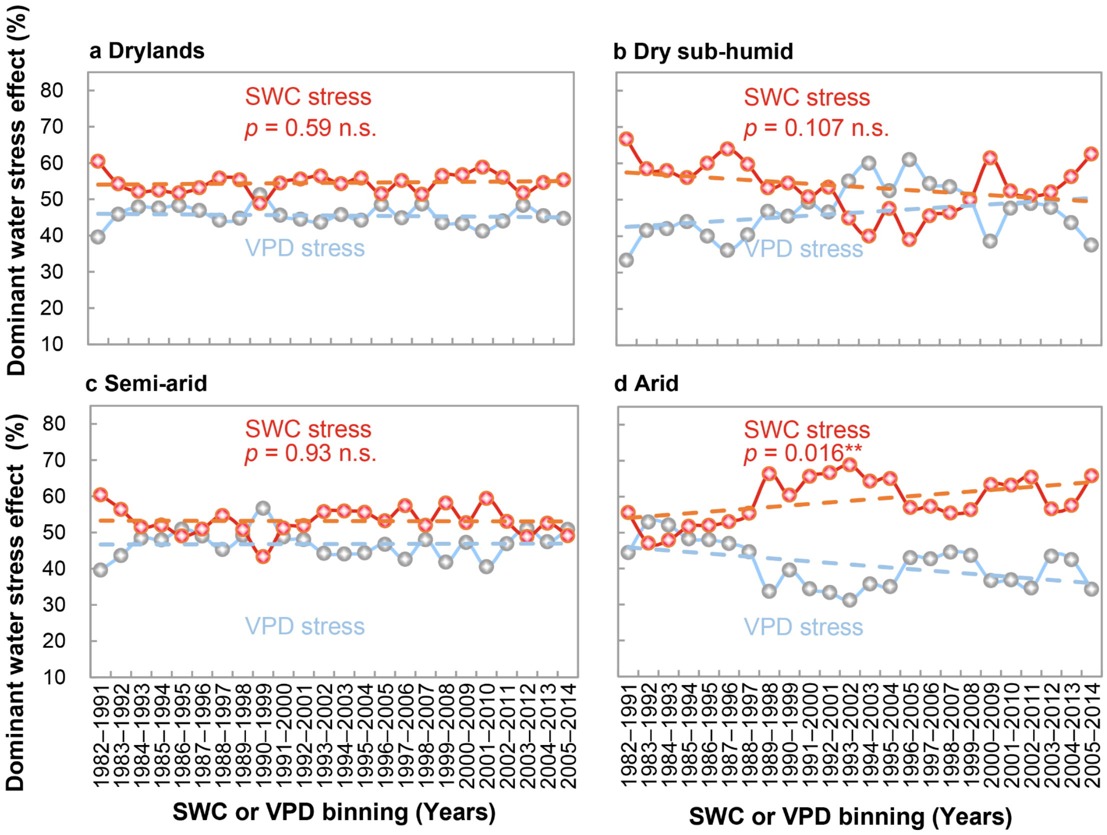

Supplementary Figure 20. Same as Fig. 5, but VPD and SWC were used as the average of multiple data, respectively.

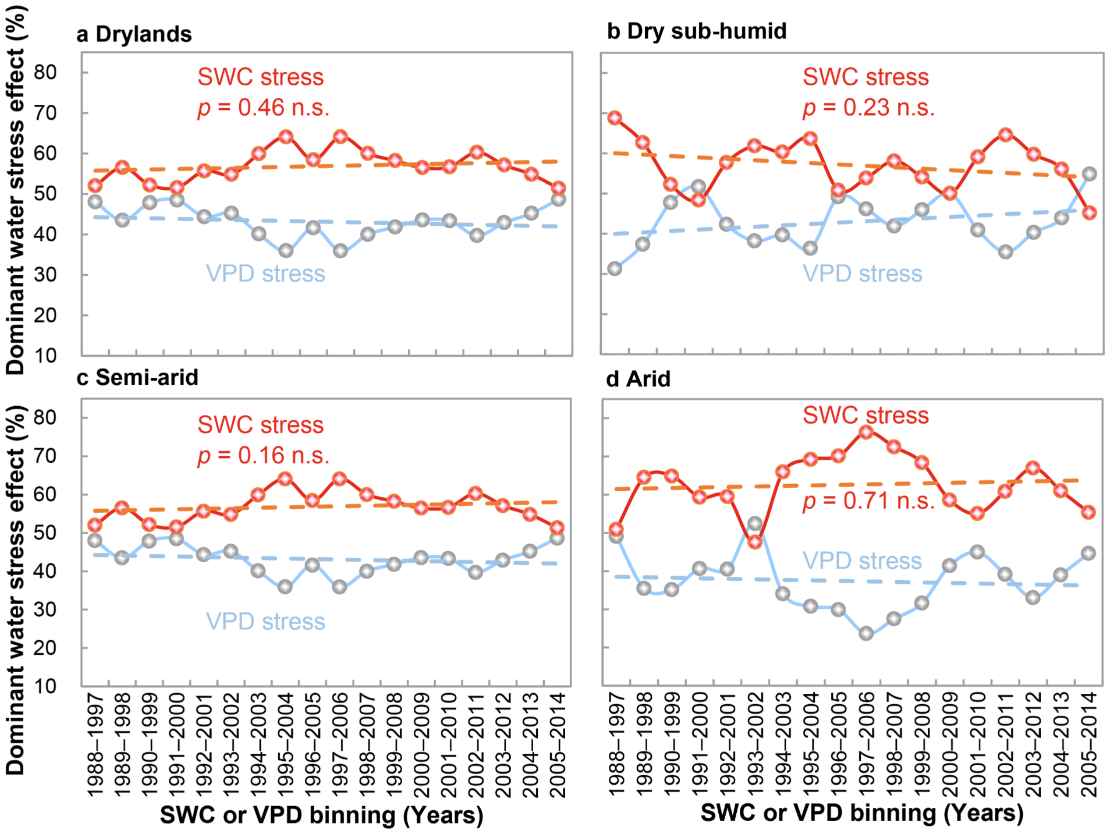

Supplementary Figure 21. Same as Supplementary Fig. 20, but vegetation optical depth (VOD) data was used as vegetation growth index.

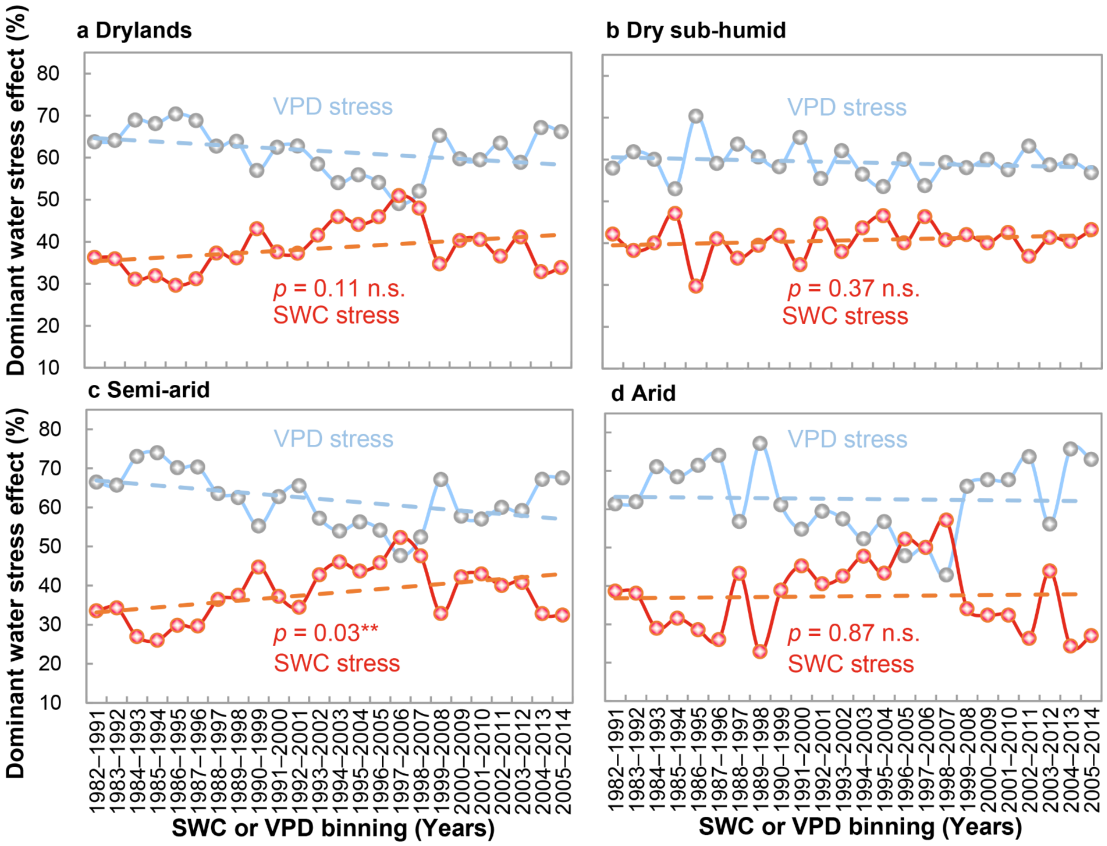

Supplementary Figure 22. Same as Supplementary Fig. 20, but gross primary production (GPP) data was used as vegetation growth index.

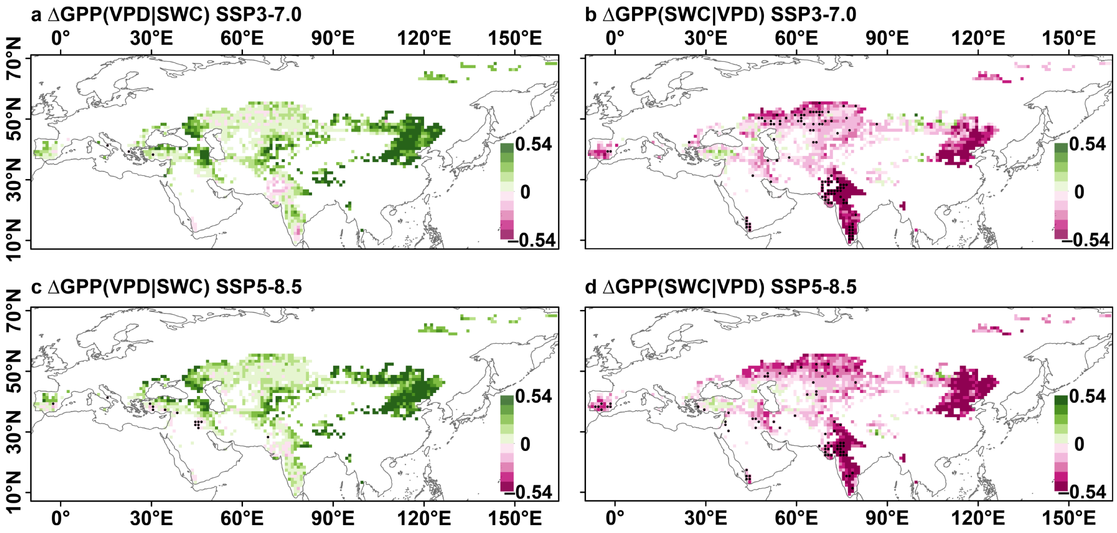

Supplementary Figure 23. Relative roles of vapor pressure deficit (VPD) and soil water content (SWC) on gross primary production (GPP) over Eurasian drylands during 2015–2100 under SSP3-7.0 (**a, b**), and SSP5-8.5 (**c, d**) scenarios. **a–d** Spatial distribution of the changes in GPP caused by high VPD (**a, c**) and low SWC (**b, d**) and dominant water stress effect (black circles; see Methods: Water-stressed ecosystems redefinitions).

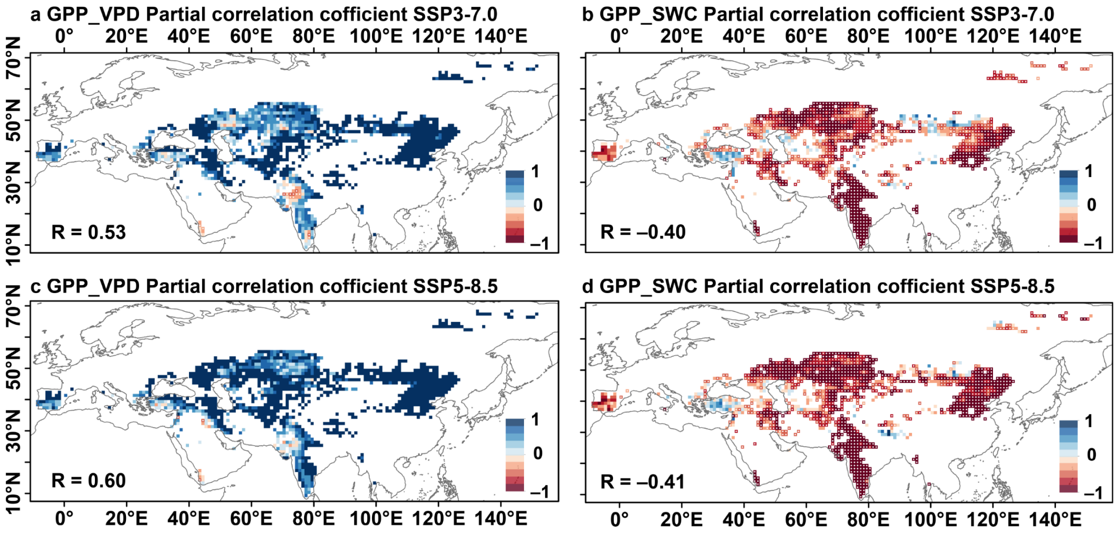

Supplementary Figure 24. Spatial patterns of the partial correlation coefficient between gross primary production (GPP) and vapor pressure deficit (VPD) (left panels) (or soil water content (SWC) (right panels)) over Eurasian drylands during 2015–2100 under SSP3-7.0 (**a, b**), and SSP5-8.5 (**c, d**) scenarios. White circles indicate significant Spearman correlations with $p < 0.05$. Considering the opposite sign between SWC and VPD, the partial correlation between GPP and SWC is displayed in number opposite to the actual value. The GPP, VPD, and SWC were used as the average of multiple data, respectively.

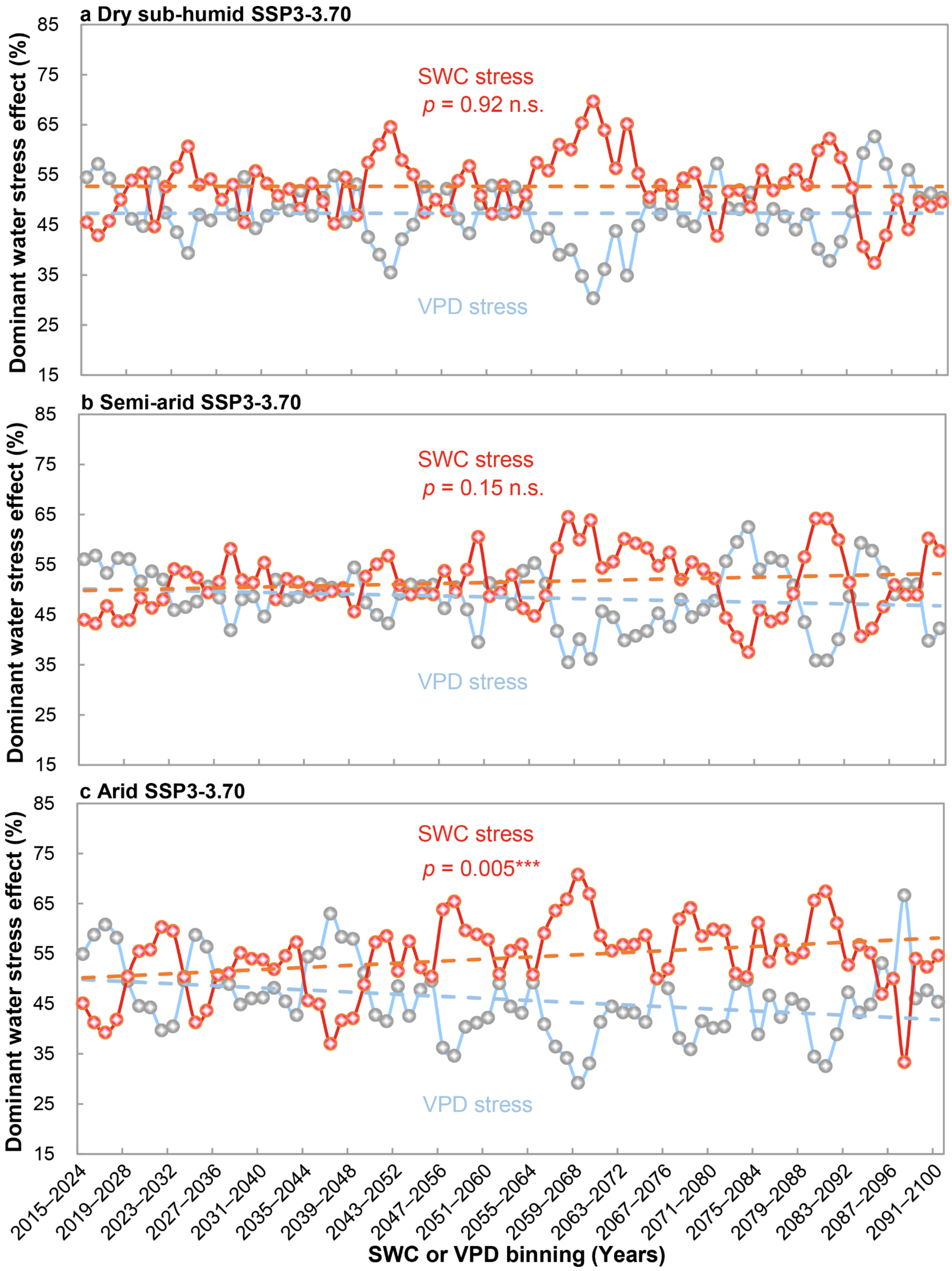

Supplementary Figure 25. Temporal dynamics of dominant water stress effect over Eurasian drylands during 2015–2100. The trends of the dominant type of water stress effect for 77 ten-year moving windows over Eurasian dry sub-humid (**a**), semi-arid (**b**), and arid (**c**) regions. The set of 11 CMIP6 ESMs for vapor pressure deficit (VPD), soil water content (SWC), and gross primary production (GPP) data during 2015–2100 under SSP3-7.0 scenarios were used. The blue and red lines represent the ecosystems dominated by VPD stress and SWC stress, respectively; SWC or VPD bins are characterized by the ten-year moving windows. Statistical significances are

shown as symbols ‘*****’, ‘****’, ‘***’, and ‘n.s.’, denoting $p < 0.001$, $p < 0.005$, $p < 0.01$, and $p > 0.1$, respectively.

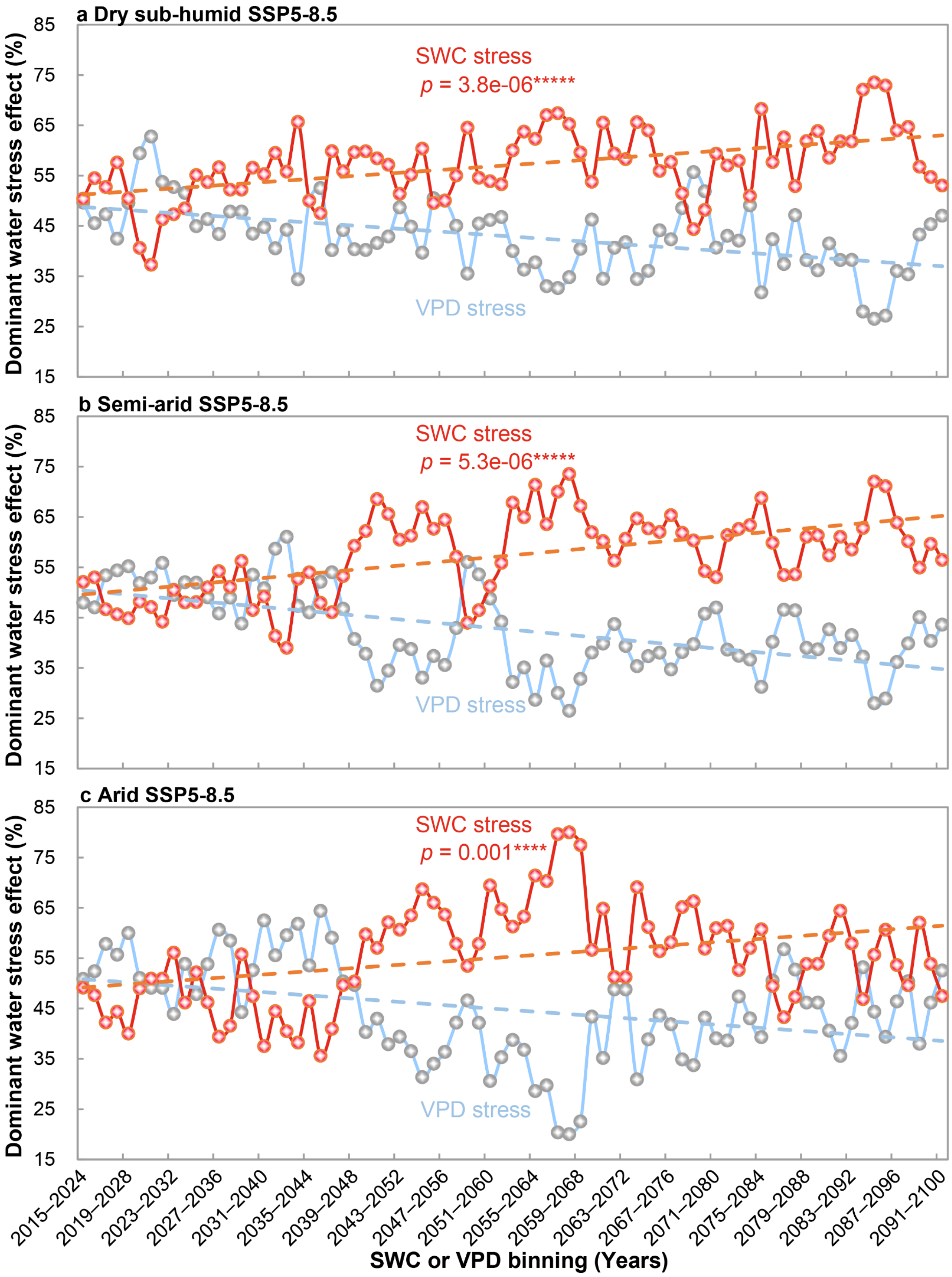

Supplementary Figure 26. Same as Supplementary Fig. 25, but VPD, SWC, and GPP data of 11CMIP6 ESMs during 2015–2100 under SSP5-8.5 scenario were used.

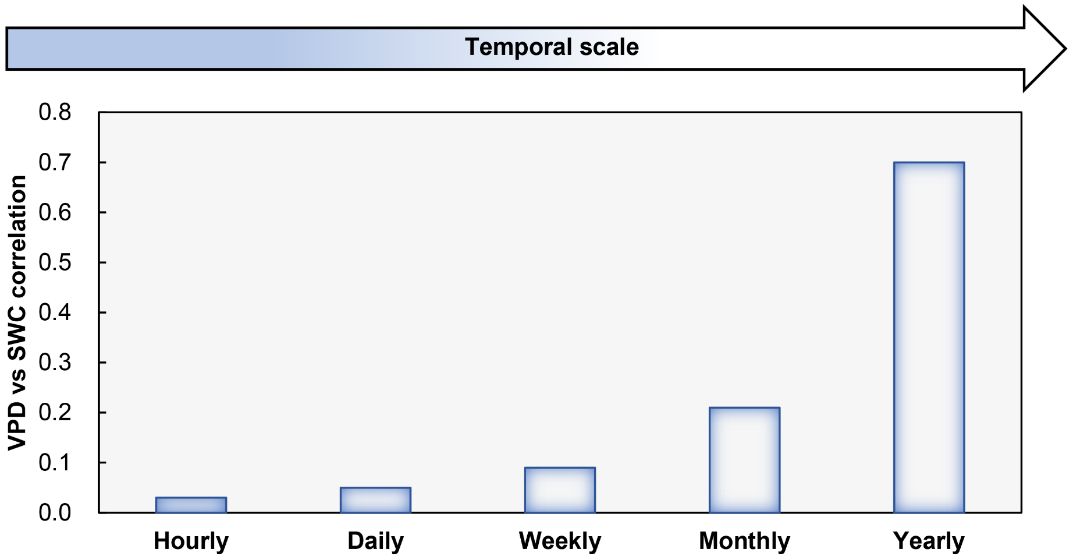

Supplementary Figure 27. Vapor pressure deficit (VPD) and soil water content (SWC) correlation at different temporal scales. VPD and SWC are weak correlation at hourly, daily, and weekly timescales, but a strong correlation at monthly and yearly timescales at Naqv site during 2012–2022.

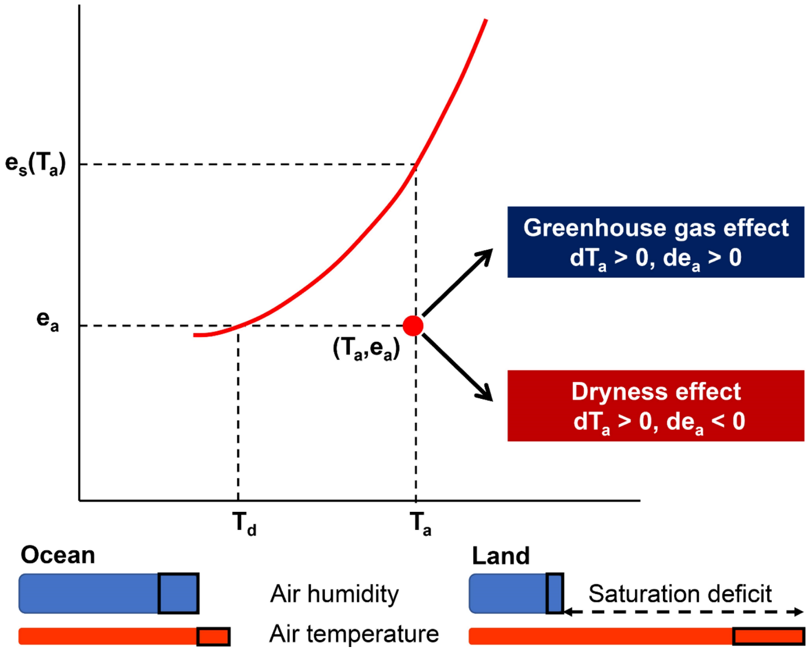

Supplementary Figure 28. The Clausius-Clapeyron curve. Atmospheric dryness effect in warmer climates, leading to expansion of atmospheric arid climate zones. The strong increases in air temperature (T_a) and consequently saturated water vapor concentration over land (e_s, red bars at lower right) exceed growth in actual water vapor concentration (e_a, blue bars). Increases in sensible and latent heat (associated, respectively, with temperature and water vapor, and represented by the area of each bar) have the same amount over land and ocean, with sensible heat increasing more over land than oceans and latent heat increasing more over oceans. Relative humidity (ratio of blue to red bar length) decreases over land [26]. T_d: dew point temperature; Red cycle: air mass; Black arrow: moving direction. In more detail, atmospheric dryness is generally regarded as a simple thermodynamic consequence of warming [27]. The greater warming occurs over drylands than the ocean [27]. e_s increases more over drylands than over oceans because e_s is driven only by temperature. It impedes the transport of moist air masses from oceans to drylands. Oceanic air masses adverted over drylands contain insufficient water vapor to keep pace with the greater increase in e_s over drylands [26,28]. Hence, the increase of near-surface specific humidity over drylands is relatively low and contains insufficient e_a to follow Clausius-Clapeyron scaling

(~7% per °C). The enlarged contrast between saturated and actual water vapor induced higher vapor pressure deficit (VPD).

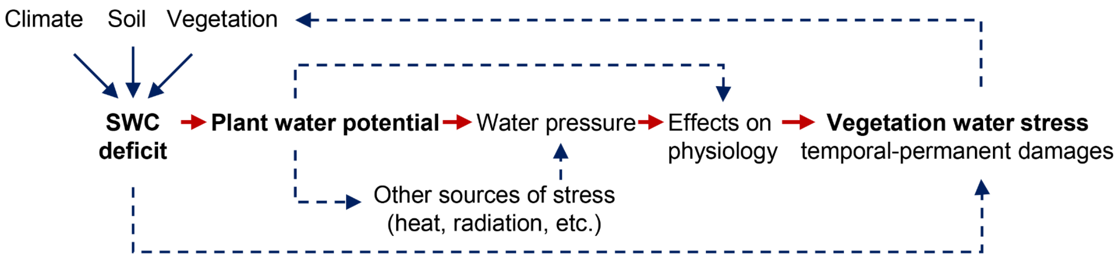

Supplementary Figure 29. A simplified scheme linking soil water content (SWC) deficit to plant water stress. The roles of climate, soil, and vegetation are linked to plant response through two fundamental processes: the first one is that SWC dynamics control the intensity and duration of SWC deficit periods, while the second one is that SWC deficit regulates plant physiology through the plant water potential, which in turn affects cell turgor and the relative water content of the plant's living cells under pressure [29].

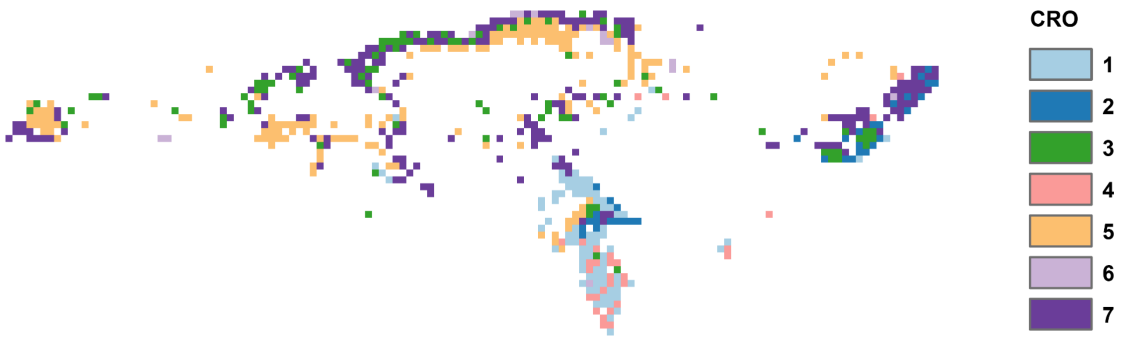

Supplementary Figure 30. Distribution of cropland classes in Eurasian drylands. The cropland (CRO) classes include: (1) irrigated wheat and rice; (2) irrigated wheat, rice, barley, and soybeans; (3) irrigated wheat, rice, cotton, and orchards; (4) rainfed wheat, rice, soybeans, sugarcane, corn, and cassava; (5) rainfed wheat and barley; (6) rainfed corn and soybeans; and (7) rainfed wheat, corn, rice, barley, and soybeans according to the Global Food Security Support Analysis Data Crop Dominance product (GFSAD1KCD). The data is available from the Land Processes Distributed Active Archive Center (https://lpdaac.usgs.gov/node/1139).

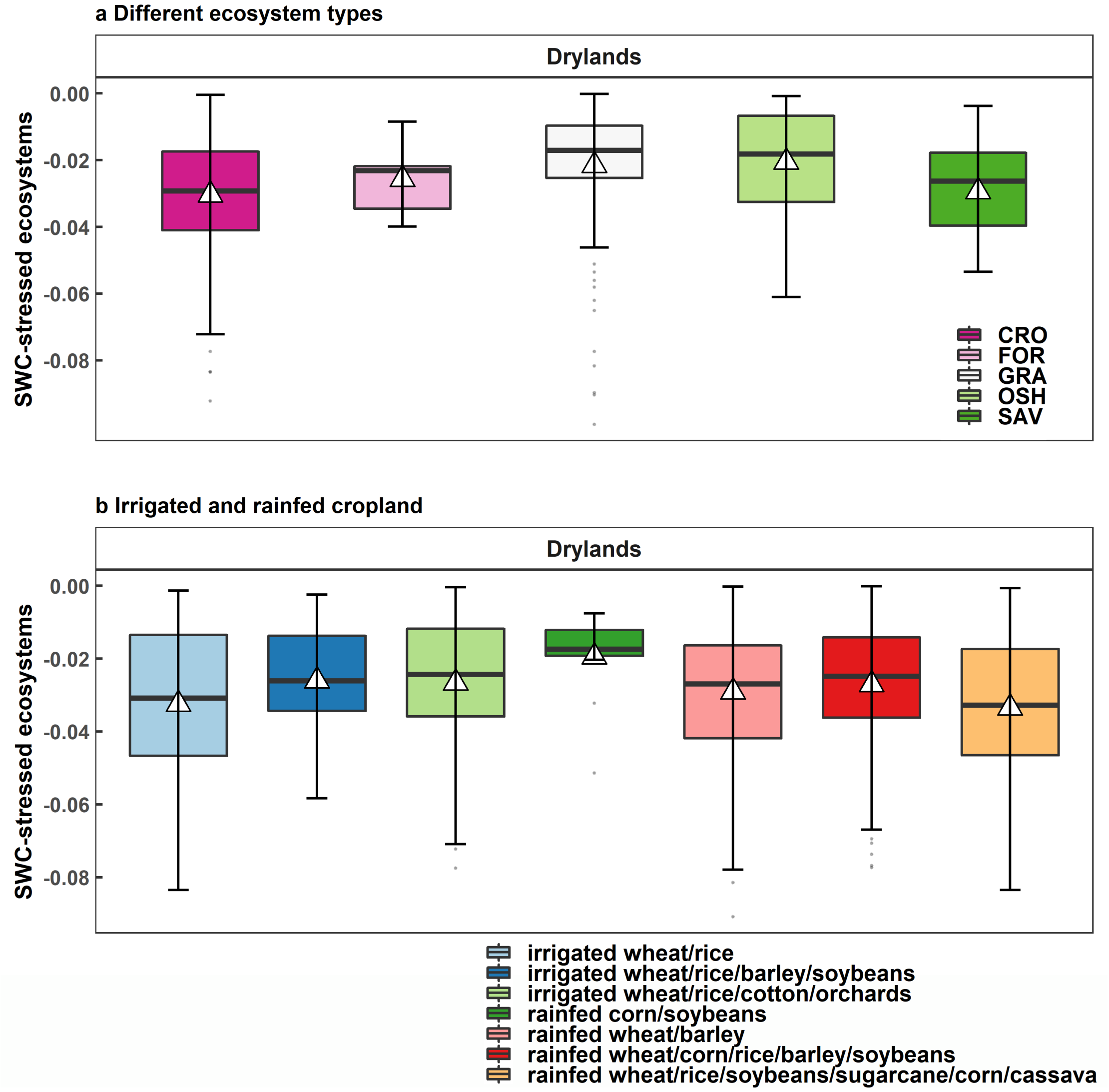

Supplementary Figure 31. Variation of soil water content (SWC) stress on different ecosystems over Eurasian drylands. SWC stress across cropland (CRO), forest (FOR), grassland (GRA), open shrublands (OSH), savannas (SAV) (**a**) following the International Geosphere-Biosphere Program based on Terra and Aqua combined MODIS Land Cover Type (MCD12Q1) Version 6, and irrigated and rained cropland (**b**) according to the Global Food Security Support Analysis Data Crop Dominance product (GFSAD1KCD). The GLEAM SWC and GIMMIS NDVI products were used.

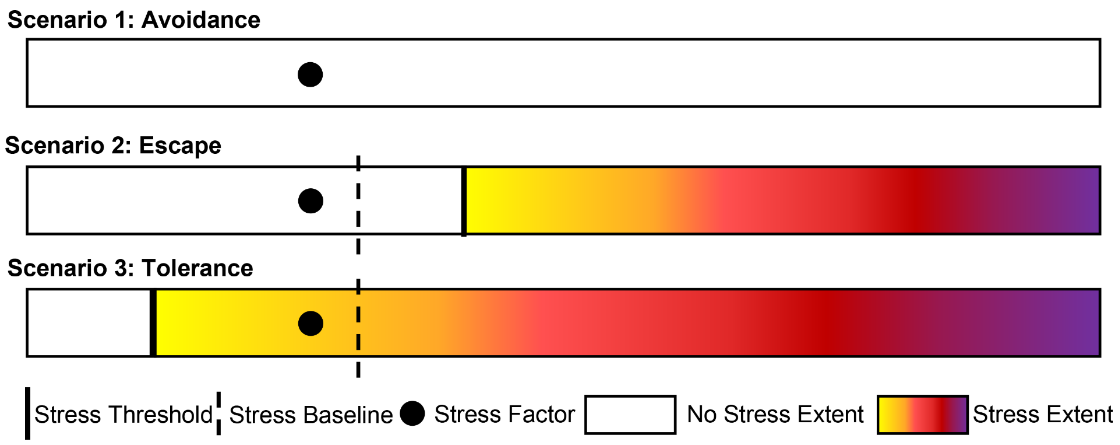

Supplementary Figure 32. Plant adaptation strategies to soil water stress under three scenarios. The adaptation strategies can be categorized as "avoidance", "escape", or "tolerance" pathways. For instance, plants can avoid soil water content (SWC) stress by increasing root biomass, root depth, and root hair, or escape future SWC stress by flowering earlier. Moreover, plants can tolerate SWC stress by adjusting their osmotic potential through physiological regulation.

Supplementary Tables

Supplementary Table 1. Comparisons between this study and others

Objective variable	Study area	Data sources	Time range	Methods	Results	References
Gross primary production (GPP) and transpiration (Tr)	A forest in south central Indiana, USA	A flux observation	2001–2013	Linear statistical model	High VPD can limit forest carbon uptake and Tr as severely as dry soil	Ref. [30]
Gs and GPP	The Corn Belt agroecosystem, USA	7 flux observations	1997–2012	Mutual information combined with wavelet analysis	High VPD dominantly controlled the canopy conductance variability	Ref. [31]
Solar-induced chlorophyll fluorescence (SIF)	Global	Satellite data, reanalysis data	2001–2016	Percentile binning	SWC was the dominant driver of dryness stress on ecosystem production across more than 70% of vegetated land areas	Ref. [7]
GPP	Naqv site, China	A flux observation, phenology observation	2012–2018	Moving time window	VPD had stronger instant inhibited effects on GPP	Ref. [32]
GPP	China	6 flux observations, satellite data	2003–2010	Partial correlation and multiple linear regression	GPP is mostly controlled by VPD in forest and shrubland and by SWC in grassland.	Ref. [33]
GPP	Europe and Global	82 flux observations, CMIP6 ESMs	1995–2018	Linear and nonlinear (Artificial neural networks) models, percentile binning	High VPD reduces photosynthesis along a large range of SWC deficits	Ref. [34]
GPP	Global	57 flux observations	1995–2014	TreeExplainer-based SHAP framework	VPD was more important for plant water stress than SWC when they removed the effect of main climatic drivers	Ref. [35]
Fluorescence quantum yield (SIFyield) and light use efficiency (LUE)	Global	40 flux observations, FLUXCOM dataset, reanalysis data	1982–2016	Percentile binning	Larger and wider impacts of VPD on ecosystem production efficiency than SWC	Ref. [36]
Normalized difference vegetation index (NDVI), vegetation optical depth (VOD), GPP	Eurasian drylands	Satellite data, reanalysis data, TRENDY DGVMs, CMIP6 ESMs, a flux observation, phenology observation	1982–2100	Percentile binning, partial correlation, moving time window	SWC stress dominated vegetation growth during 1982–2014 and the dominance of SWC stress were projected to further increase with future warming	This study

Supplementary Table 2. Details of vegetation growth, vapor pressure deficit, soil water content, precipitation, and potential evaporation datasets were used in this study.

Variable	Data source	Resolution (degree)	Temporal coverage
Vegetation growth indices	GIMMS NDVI3g	0.08 × 0.08	1982–2015
	VODCA_Ku-band	0.25 × 0.25	1988–2017
	GPP_{NIRv}	0.05 × 0.05	1982-2018
Vapor pressure deficit	TERRACLIMATE	0.04 × 0.04	1958–2020
	ERA-Interim	0.125 × 0.125	1979–2019
	ERA5.Land	0.1 × 0.1	1979–2020
	MERRA-2	0.667 × 0.667	1980–2016
Soil water content	GLDAS_NOAH025_M.020	0.25 × 0.25	1948–2014
	GLEAMv3.5a	0.25 × 0.25	1980–2020
Precipitation	CRU.TS4.05	0.5 × 0.5	1901–2020
	PGFv3	0.25 × 0.25	1948–2016
	UDelv5.01	0.5 × 0.5	1900–2017
	ERA5.Land	0.1 × 0.1	1981–2020
	MSWEPv2.8	0.1 × 0.1	1979–2020
Potential evaporation	GLDAS_NOAH025_M.020	0.25 × 0.25	1948–2014
	NCEP.NCAR	1.875 × 1.875	1948–2020
	PGFv3	0.25 × 0.25	1948–2016

Supplementary Table 3. Details of the 18 terrestrial ecosystem models from the TRENDY-v9 DGVMs were used in this study.

	Model name	Abbreviation	Resolution (degree)
1	Canadian Land Surface Scheme Including Biogeochemical Cycles	CLASSIC	2.789 × 2.789
2	Community Land Model version 4.5	CLM5.0	1.25 × 0.9375
3	Dynamic Land Ecosystem Model	DLEM	0.5 × 0.5
4	Integrated BIosphere Simulator	IBIS	1 × 1
5	Integrated Science Assessment Model	ISAM	0.5 × 0.5
6	Interactions between Soil, Biosphere and Atmosphere-Carbon Transfer by Reservoir Interactions and Percolation	ISBA_CTRIP	1 × 1
7	Jena Scheme for Biosphere-Atmosphere Coupling in Hamburg	JSBACH	1.875 × 1.875
8	The Joint UK Land Environment Simulator	JULE-ES-1.0	1.875 × 1.25
9	Lund-Potsdam-Jena Dynamic Global Vegetation Model	LPJ	0.5 × 0.5
10	Lund-Potsdam-Jena General Ecosystem Simulator	LPJ-GUESS	0.5 × 0.5
11	Exchanges model of the University of Bern	LPX-Bern	0.5 × 0.5
12	Organising Carbon in Nature	OCN	1 × 1
13	Organizing Carbon and Hydrology in Dynamic Ecosystems	ORCHIDEE	0.5 × 0.5
14	Organising Carbon and Hydrology In Dynamic Ecosystems-Carbon-Nitrogen-Phosphorus	ORCHIDEE-CNP	2 × 2
15	Organizing Carbon and Hydrology in Dynamic Ecosystems v3	ORCHIDEEv3	2 × 2
16	Sheffield Dynamic Global Vegetation Model	SDGVM	1 × 1
17	Vegetation Integrative SImulator for Trace gases	VISIT	0.5 × 0.5
18	Yale Interactive terrestrial Biosphere model	YIBs	1 × 1

Supplementary Table 4. Details of the 11 Coupled Model Intercomparison Project Phase 6 (CMIP6) Earth system models (ESMs) were used in this study.

	ESMs	Institution ID	Institution	Resolution (degree)
1	ACCESS-ESM1-5	CSIRO	Commonwealth Scientific and Industrial Research Organization and Bureau of Meteorology, Australia	1.2414 × 1.8750
2	CESM2-WACCM	NCAR	National Center for Atmospheric Research, Boulder, USA	0.94 × 1.25
3	CanESM5	CCCma	Canadian Center for Climate Modeling and Analysis, Canada	2.8125 × 2.8125
4	EC-Earth3-Veg	EC-Earth-Consortium	EC-Earth-Consortium, EU	0.70 × 0.70
5	INM-CM4-8	INM-CM	Institute for Numerical Mathematics (INM), Russia	1.5 × 2.0
6	INM-CM5-0	INM-CM	Institute for Numerical Mathematics, Russia	1.5 × 2.0
7	IPSL-CM6A-LR	IPSL	Institute Pierre Simon Laplace, France	1.26 × 2.50
8	MPI-ESM1-2-HR	MPI-M	Max Planck Institute for Meteorology, Germany	0.94 × 0.94
9	MPI-ESM1-2-LR	MPI-M	Max Planck Institute for Meteorology, Germany	0.94 × 0.94
10	NorESM2-LM	NCC	Norwegian Climate Center, Norway	1.875 × 2.5
11	NorESM2-MM	NCC	Norwegian Climate Center, Norway	0.94 × 1.25

Supplementary Table 5. Relative roles of vapor pressure deficit (VPD) and soil water content (SWC) on gross primary production (GPP) during the growing season, at Naqv alpine meadow ecosystem (AME) station from 2012 to 2022.

	Hourly	Daily	Weekly	Monthly	Yearly
Non-VPD-stressed AME	✓				
Non-SWC-stressed AME					
VPD-stressed AME		✓	✓	✓	✓
SWC-stressed AME	✓	✓	✓	✓	✓
AME dominated by VPD stress					
AME dominated by SWC stress	✓	✓	✓	✓	✓

Supplementary Table 6. Relative roles of vapor pressure deficit (VPD) and soil water content (SWC) on gross primary production (GPP) at hourly intervals during the growing season, at Naqv alpine meadow ecosystem (AME) station from 2012 to 2022.

	7:00	8:00	9:00	10:00	11:00	12:00	13:00	14:00	15:00	16:00	17:00	18:00	19:00	20:00
Non-VPD-stressed AME	✓	✓		✓	✓				✓	✓	✓	✓	✓	✓
Non-SWC-stressed AME								✓				✓		
VPD-stressed AME			✓			✓	✓	✓						
SWC-stressed AME	✓	✓	✓	✓	✓	✓	✓		✓	✓	✓		✓	✓
AME dominated by VPD stress								✓						
AME dominated by SWC stress	✓	✓	✓	✓	✓	✓	✓		✓	✓	✓		✓	✓

Supplementary References

1. Tucker CJ, Pinzon JE, Brown ME *et al.* An extended AVHRR 8-km NDVI dataset compatible with MODIS and SPOT vegetation NDVI data. *Int J Remote Sens* 2005; **26**: 4485–98.

2. Pinzon JE, Tucker CJ. A non-stationary 1981-2012 AVHRR NDVI3g time series. *Remote Sens* 2014; **6**: 6929–60.

3. Moesinger L, Dorigo W, de Jeu R *et al.* The global long-term microwave Vegetation Optical Depth Climate Archive (VODCA). *Earth Syst Sci Data* 2020; **12**: 177–96.

4. Wang S, Zhang Y, Ju W *et al.* Tracking the seasonal and inter-annual variations of global gross primary production during last four decades using satellite near-infrared reflectance data. *Sci Total Environ* 2021; **755**: 142569.

5. Huang M, Piao S, Janssens IA *et al.* Velocity of change in vegetation productivity over northern high latitudes. *Nat Ecol Evol* 2017; **1**: 1649–54.

6. Sulla-Menashe D, Gray JM, Abercrombie SP *et al.* Hierarchical mapping of annual global land cover 2001 to present: The MODIS Collection 6 Land Cover product. *Remote Sens Environ* 2019; **222**, 183–94.

7. Liu L, Gudmundsson L, Hauser M *et al.* Soil moisture dominates dryness stress on ecosystem production globally. *Nat Commun* 2020; **11**: 1–9.

8. Abatzoglou JT, Dobrowski SZ, Parks SA *et al.* TerraClimate, a high-resolution global dataset of monthly climate and climatic water balance from 1958-2015. *Sci Data* 2018; **5**: 1–12.

9. Dee DP, Uppala SM, Simmons AJ *et al.* The ERA-Interim reanalysis: Configuration and performance of the data assimilation system. *Q. J. R. Meteorol. Soc.* 2011; **137**: 553–97.

10. Gelaro R, McCarty W, Suárez MJ *et al.* The modern-era retrospective analysis for research and

applications, version 2 (MERRA-2). *J Clim* 2017; **30**: 5419–54.

11. Muñoz-Sabater J, Dutra E, Agustí-Panareda A *et al.* ERA5-Land: A state-of-the-art global reanalysis dataset for land applications. *Earth Syst Sci Data* 2021; **13**: 4349–83.

12. Qing Y, Wang S, Ancell BC *et al.* Accelerating flash droughts induced by the joint influence of soil moisture depletion and atmospheric aridity. *Nat Commun* 2022; **13**: 1–10.

13. Miralles DG, de Jeu RAM, Gash JH *et al.* Magnitude and variability of land evaporation and its components at the global scale. *Hydrol Earth Syst Sci* 2011; **15**: 967–81.

14. Miralles DG, Holmes TRH, de Jeu RAM *et al.* Global land-surface evaporation estimated from satellite-based observations. *Hydrol Earth Syst Sci* 2011; **15**: 453–69.

15. Martens B, Miralles DG, Lievens H *et al.* GLEAM v3: Satellite-based land evaporation and root-zone soil moisture. *Geosci Model Dev* 2017; **10**: 1903.

16. Rui HL, Beaudoing H. README document for NASA GLDAS version 2 data products. *Goddard Earth Sciences Data and Information Services Center (GES DISC)* 2020; **16**.

17. Lian X, Piao S, Chen A *et al.* Multifaceted characteristics of dryland aridity changes in a warming world. *Nat Rev Earth Environ* 2021; **2**: 232–50.

18. Harris I, Osborn TJ, Jones P *et al.* CRU TS4.05: Climatic Research Unit (CRU) Time-Series (TS) version 4.05 of high-resolution gridded data of month-by-month variation in climate (Jan. 1901- Dec. 2020). *Sci Data* 2020; **7**.

19. Sheffield J, Goteti G, Wood EF. Development of a 50-year high-resolution global dataset of meteorological forcings for land surface modeling. *J Clim* 2006; **19**: 3088–111.

20. Willmott CJ, Matsuura K. Terrestrial air temperature and precipitation: monthly and annual time series (1900–2017).

http://climate.geog.udel.edu/~climate/html_pages/README.ghcn_ts2.html 2018.

21. Kistler R, Kalnay E, Collins W *et al.* The NCEP-NCAR 50-year reanalysis: Monthly means CD-ROM and documentation. *Bull Am Meteorol Soc* 2001; **82**: 247–68.

22. Zhang T, Tang Y, Xu M *et al.* Joint control of alpine meadow productivity by plant phenology and photosynthetic capacity. *Agric For Meteorol* 2022; **325**: 109135.

23. Barrow CJ. World atlas of desertification (United Nations Environment Programme). Edited by N. Middleton and D. S. G. Thomas. Edward Arnold, London, *Land Degrad Dev*, 1992; **3**: 69.

24. UNEP, 1997. World atlas of desertification 2ED. United Nations Environment Programme.

25. Cherlet M, Hutchinson C, Reynolds J *et al.* World Atlas of Desertification: Rethinking Land Degradation and Sustainable. World Atlas of Desertification 2018.

26. Sherwood S, Fu Q. A drier future? *Science* 2014; **343**: 737–9.

27. Intergovernmental Panel on Climate Change (IPCC). Climate Change 2021: The Physical Science Basis. *IPCC Cambridge Univ Press Ch11* 2021.

28. Byrne MP, O'Gorman PA. Trends in continental temperature and humidity directly linked to ocean warming. *Proc Natl Acad Sci USA* 2018; **115**: 4863–8.

29. Rodriguez-Iturbe I, Porporato A. Ecohydrology of water-controlled ecosystems - soil moisture and plant dynamics. *Cambridge Univ. Press* 2004.

30. Sulman BN, Roman DT, Yi K *et al.* High atmospheric demand for water can limit forest carbon uptake and transpiration as severely as dry soil. *Geophys Res Lett* 2016; **43**: 9686–95.

31. Kimm H, Guan K, Gentine P *et al.* Redefining droughts for the U.S. Corn Belt: The dominant role of atmospheric vapor pressure deficit over soil moisture in regulating stomatal behavior of

Maize and Soybean. *Agric For Meteorol* 2020; **287**: 107930.

32. Xu M, Zhang T, Zhang Y *et al.* Drought limits alpine meadow productivity in northern Tibet. *Agric For Meteorol* 2021; **303**: 108371.

33. Chen N, Song C, Xu X *et al.* Divergent impacts of atmospheric water demand on gross primary productivity in three typical ecosystems in China. *Agric For Meteorol* 2021; **307**: 108527.

34. Fu Z, Ciais P, Prentice IC *et al.* Atmospheric dryness reduces photosynthesis along a large range of soil water deficits. *Nat Commun* 2022; **13**: 1–10.

35. Wang H, Yan S, Ciais P *et al.* Exploring complex water stress–gross primary production relationships: Impact of climatic drivers, main effects, and interactive effects. *Glob Chang Biol* 2022; **28**: 4110–23.

36. Lu H, Qin Z, Lin S *et al.* Large influence of atmospheric vapor pressure deficit on ecosystem production efficiency. *Nat Commun* 2022; **13**: 10–3.
